# Stroke and cognitive decline: associations of social support and family connections in CHARLS and ELSA cohorts

**DOI:** 10.1186/s12889-026-28890-0

**Published:** 2026-08-06

**Authors:** Mingdong Li, Kewei Xiao, Chaoli Song, Shengrong Long

**Affiliations:** 1 Department of Thoracic Surgery, Tongji Hospital, Tongji Medical College, Huazhong University of Science and Technology, Wuhan, Hubei Province 430030 China; 2 Department of Neurosurgery, Western Theater Command Air Force Hospital, Chengdu, Sichuan Province 6000211 China

**Keywords:** Stroke, Cognitive Decline, Social Support, Family Connections, CHARLS, ELSA

## Abstract

**Background:**

Stroke is associated with accelerated cognitive decline in older adults, but whether social support and family connections statistically moderate this association has rarely been tested formally in longitudinal, cross-national data.

**Methods:**

We analyzed the China Health and Retirement Longitudinal Study (CHARLS; *N* = 11,569; Wave 1 [2011] baseline to Wave 4 [2018], up to 7.2 years) and the English Longitudinal Study of Ageing (ELSA; *N* = 5,713; Wave 3 [2006–2007] baseline to Wave 9 [2019], up to 13.0 years). Cognitive function was assessed with a harmonized composite (orientation, immediate and delayed word recall, plus pentagon drawing in CHARLS). Linear mixed-effects models with random intercepts and slopes estimated the incident-stroke × time interaction (annual cognitive decline, points/year), adjusting for age, sex, BMI, education, marital status, smoking, drinking, comorbidity count, and residence (CHARLS only). A three-way interaction (stroke × social support/family connection × time) formally tested moderation. Propensity-score matching (1:2 nearest-neighbor) and subgroup analyses (age, sex, education) served as sensitivity analyses.

**Results:**

Incident stroke occurred in 770 CHARLS (6.6%) and 347 ELSA (6.1%) participants. In fully adjusted models, stroke was associated with significantly faster cognitive decline in both cohorts (CHARLS: β = −0.068 points/year, 95% CI − 0.123 to − 0.013, *p* = 0.016; ELSA: β = −0.190, 95% CI − 0.229 to − 0.152, *p* < 0.001); after propensity-score matching, the ELSA association remained robust (β = −0.150, *p* < 0.001) while the CHARLS estimate was attenuated and no longer significant (β = −0.029, *p* = 0.386). The formal three-way interaction showed that higher family connection significantly attenuated stroke-related decline in ELSA ; the corresponding interaction for social support in CHARLS was directionally consistent but not significant (β = +0.082, 95% CI − 0.037 to 0.202, *p* = 0.177). Subgroup analyses showed a significant stroke-decline association across all age, sex, and education strata in ELSA, and only among male and less-educated participants in CHARLS.

**Conclusion:**

Incident stroke was associated with faster cognitive decline in both a Chinese and an English cohort of middle-aged and older adults.

A formal statistical test indicated that family connections significantly moderated this association in ELSA, whereas a similar but non-significant pattern was observed for social support in CHARLS.

These associations warrant further investigation of social relationships as a potentially modifiable factor in post-stroke cognitive health; the observational design precludes causal conclusions.

**Supplementary Information:**

The online version contains supplementary material available at https://doi.org/10.1186/s12889-026-28890-0.

## Introduction

### Global burden of stroke and cognitive impairment

Cognitive decline represents a critical public health challenge, fundamentally impacting independence, quality of life, and healthcare systems among older adults worldwide. Stroke, as a leading cerebrovascular disorder, continues to escalate globally, with projections indicating a 50% increase in stroke-related deaths between 2020 and 2050, rising from 6.6 million to 9.7 million annually [1, 2]. Currently, stroke remains the second leading cause of death and the third leading cause of death and disability combined globally, with 77% of prevalent strokes, 87% of fatal strokes, and 89% of disability-adjusted life years occurring in low- to middle-income countries [2, 3].

Beyond immediate mortality, stroke is a primary contributor to long-term disability and dementia worldwide [2, 4]. Approximately 795,000 people experience stroke annually in the United States alone, with survivors often facing profound neurological deficits including impairments in movement, language, and cognition [5]. The economic burden is substantial, with stroke-associated costs reaching $56.2 billion between 2019 and 2020 in the United States [5].

### Post-stroke cognitive impairment: prevalence and mechanisms

PSCI (Post-stroke cognitive impairment) affects a substantial proportion of stroke survivors, with prevalence rates varying by assessment method, timing, and population. Systematic reviews indicate cognitive impairment affects 41.4% to 70% of stroke survivors, with approximately 30% developing PSCI within three months post-stroke [6–9]. A recent large-scale retrospective study reported PSCI incidence of 55.18% among first-time stroke patients [10]. This variability in prevalence estimates reflects heterogeneity in definitions, measurement tools, and population characteristics [11, 12]. Critically, nearly half of individuals with PSCI progress to post-stroke dementia within five years, associated with increased mortality and reduced median survival [6].

The mechanisms underlying PSCI are multifactorial. Stroke location critically determines specific cognitive deficits, with frontal lobe infarcts associated with executive dysfunction and temporal lesions with memory impairment [8, 13]. Multi-territorial and extensive infarcts substantially increase cognitive impairment risk [14]. Vascular risk factors—including hypertension, diabetes, atrial fibrillation, and hyperlipidemia—contribute through progressive vascular pathology that damages brain structures and impairs cerebral blood flow [8, 15]. Recent research identified additional predictors including age ≥ 60 years (OR = 6.81), elevated BMI (OR = 2.69–6.53), specific lesion locations (OR = 6.04–6.20), white matter changes (OR = 3.18), and depression (OR = 1.09) [10]. Manifestations of cerebral small vessel disease, including covert infarcts, white matter lesions, and microbleeds, further compound cognitive outcomes [16].

### Cognitive trajectories before and after stroke

Longitudinal research demonstrates that cognitive decline often begins before clinical stroke and accelerates substantially afterward [17, 18]. Analysis of 14 population cohorts revealed marked cognitive trajectory decline both pre- and post-stroke, suggesting stroke represents both a consequence and accelerator of underlying cerebrovascular pathology [18]. While most rapid recovery occurs within three to four months post-stroke, cognitive trajectories show substantial heterogeneity [19]. Approximately 26% of patients demonstrate cognitive improvement within three months [20], yet cognitive impairment persists in many patients despite acute interventions, with 33.3% of intravenous thrombolysis recipients and 50% of endovascular thrombectomy recipients exhibiting impairment at six-year follow-up [9].

### Associations of social support and family connections with cognitive decline

Emerging evidence emphasizes the critical role of psychosocial factors in cognitive health among older adults. Social support, defined as support accessible through social ties to individuals, groups, and communities, has been consistently associated with better cognitive outcomes [21, 22]. A systematic review of 85 studies demonstrated that functional social support, particularly emotional support, is associated with higher cognitive function in middle-aged and older adults [22].

Social support appears to protect against cognitive decline through multiple mechanisms. The stress buffer hypothesis posits that social support reduces negative reactions to stressful experiences, decreases inflammatory responses, and promotes healthy behaviors, thereby benefiting cognitive function [23]. Perceived social support provides emotional experiences of respect and understanding within social networks, demonstrating positive effects on cognition [23, 24]. A meta-analysis examining studies from 2012 to 2020 found poor social relationships significantly associated with cognitive decline, with odds ratios ranging from 1.11 to 1.15 depending on support type [25]. Large cohort data from the Chinese Longitudinal Healthy Longevity Survey revealed that social support, particularly children’s visits, consistently associated with lower cognitive impairment incidence [21].

Family connections represent particularly potent forms of social engagement for stroke survivors. Family members, especially spouses, provide crucial instrumental and emotional support significantly impacting rehabilitation outcomes [26, 27]. Research demonstrates that family support positively influences functional status improvement in first-stroke patients, particularly those with severe impairments [26]. Recent randomized trials showed family-based intervention programs significantly improved family function, decreased caregiver burden, and enhanced functional status while reducing depression in stroke survivors [28]. Most stroke survivors receive home care from family caregivers with limited formal training, and families actively involved in rehabilitation feel more prepared for caregiving responsibilities [29, 30]. However, 54% of families experience relationship problems post-stroke, highlighting the complex dynamics of family adaptation [31].

### Knowledge gaps and study objectives

Despite growing recognition of psychosocial factors in cognitive health, the extent to which social support and family connections are associated with stroke-related cognitive decline remains incompletely understood, particularly across diverse cultural contexts. Most research has focused on individual risk factors without comprehensively examining social dynamics and their interactions with post-stroke cognitive decline [32, 33]. Cross-national comparative studies remain limited.

The China Health and Retirement Longitudinal Study (CHARLS) and English Longitudinal Study of Ageing (ELSA) represent well-established cohorts capturing comprehensive data on cognitive function, social networks, and family relationships across culturally distinct populations, providing optimal opportunities to examine cognitive trajectories and identify modifiable protective factors [34, 35].

We hypothesized that stroke would be associated with faster cognitive decline in both cohorts, and that among stroke survivors, higher levels of social support and family connection would be associated with slower cognitive decline. We selected China and England for comparison because they represent distinct sociocultural contexts of aging and later-life support. In China, family-based care and intergenerational support remain central features of older adults’ social lives, whereas in England, social support may be shaped more by independent living arrangements and broader community or peer-based networks. By examining the CHARLS and ELSA cohorts separately, we aimed to explore whether similar patterns could be observed across these different settings. Given differences in social structure, culture, and measurement, we considered that the strength and pattern of these associations might vary across cohorts. This study aims to: (1) investigate associations between stroke and cognitive decline in two large, culturally diverse longitudinal cohorts; (2) examine whether social support (CHARLS) and family connection (ELSA) moderate the association between incident stroke and cognitive decline. Understanding these relationships may inform evidence-based strategies to preserve cognitive function and improve quality of life following stroke, with implications for rehabilitation programs and public health interventions targeting social engagement.

## Materials and methods

### Data sources

This study utilized data from two large-scale longitudinal aging cohorts. The China Health and Retirement Longitudinal Study (CHARLS; http://charls.pku.edu.cn) is a nationally representative survey of Chinese residents aged 45 years and older, collecting information on health, socioeconomic status, and social networks biennially from 2011. The English Longitudinal Study of Ageing (ELSA; UK Data Service, https://ukdataservice.ac.uk) surveys English adults aged 50 years and older with repeated assessments from 2002. Both cohorts include standardized cognitive assessments, sociodemographic information, health status, and psychosocial measures, enabling cross-national comparison. All data were obtained from the official study websites. The current analysis used harmonized data from the Gateway to Global Aging Data repository.

### Participant selection

In CHARLS, Wave 1 (2011) served as the baseline, with follow-up assessments at Waves 2 (2013), 3 (2015), and 4 (2018), providing up to 7.2 years of follow-up. In ELSA, Wave 3 (2006–2007) served as the baseline, with follow-up assessments at Waves 4 (2008–2009), 5 (2010–2011), 6 (2012–2013), 7 (2014–2015), 8 (2016–2017), and 9 (2018–2019), yielding up to 13.0 years of follow-up.

Participants were included if they met the age eligibility threshold of each cohort (≥ 45 years for CHARLS; ≥50 years for ELSA), had no self-reported stroke at baseline, had valid body mass index (BMI) measurements (10–60 kg/m² for CHARLS; for ELSA, BMI was derived from Wave 2 or Wave 4 nurse visits since measured BMI is only available at even-numbered waves), had cognitive data at baseline and at least one follow-up wave, and had complete data on all baseline covariates. The stepwise exclusion process for each cohort is reported in Supplementary Table S1 and S2.

### Cognitive function assessment

Cognitive function was assessed using a harmonized composite score derived from components available across all waves in both cohorts. To ensure cross-wave and cross-cohort comparability, we excluded cognitive tests not consistently available: specifically, the serial 7s subtraction test was excluded because it is only available from Wave 7 onward in ELSA, and the drawing test was included only for CHARLS as ELSA does not have a comparable measure.

The cognitive composite score was calculated as the sum of the following components at each wave. Orientation, ranging from 0 to 4 points, assessed the ability to correctly state the current day of week, day of month, month, and year, corresponding to the harmonized variable r{w}orient in both cohorts. Immediate word recall, ranging from 0 to 10 points, measured the number of words correctly recalled immediately after a 10-word list was read aloud, corresponding to r{w}imrc. Delayed word recall, ranging from 0 to 10 points, measured the number of words correctly recalled after a delay of approximately four to five minutes, corresponding to r{w}dlrc. The drawing test, ranging from 0 to 1 point and available only in CHARLS, assessed the ability to copy intersecting pentagons, corresponding to r{w}draw. The total cognitive score ranged from 0 to 25 in CHARLS and 0 to 24 in ELSA, with higher scores indicating better cognitive function. Participants missing any component of the cognitive score at a given wave were excluded from that wave’s observation. All cognitive components used in the composite score were verified to be available across all waves included in the analysis for each cohort: CHARLS Waves 1 through 4 (r{1–4}orient, r{1–4}imrc, r{1–4}dlrc, r{1–4}draw) and ELSA Waves 3 through 9 (r{3–9}orient, r{3–9}imrc, r{3–9}dlrc).

### Social support and family contact

Social support and family contact were assessed as key factors potentially influencing cognitive decline. In CHARLS, social support from adult children was assessed across three dimensions following Xie et al. (2024) [36]. Emotional support was measured by the frequency of face-to-face contact with children (h1kcntf: any weekly in-person contact with children). Financial support was assessed by whether participants had received any financial transfers from children or grandchildren in the past year (h1fcany). Instrumental support was measured by whether participants received informal care from children or grandchildren for activities of daily living or instrumental activities of daily living (r1rccare). Each dimension was coded as a binary variable (0 = absent, 1 = present). A composite social support score ranging from 0 to 3 was derived by summing the three binary dimensions and dichotomized at the sample median, with scores of 2 or above categorized as “high support” and scores below 2 categorized as “low support.”

In ELSA, family connections were assessed using frequency-of-contact items from the Social Networks module at Wave 3 (baseline). Four contact dimensions were assessed: any weekly in-person contact with children (r3kcntf), any weekly telephone or email contact with children (r3kcntpm), any weekly in-person contact with friends (r3fcntf), and any weekly in-person contact with relatives (r3rcntf). Each dimension was coded as a binary variable (0 = no weekly contact, 1 = any weekly contact). A composite family contact score ranging from 0 to 4 was derived by summing the four binary dimensions and dichotomized at the sample median, with scores of 2 or above categorized as “high connection” and scores below 2 categorized as “low connection.”

Although the specific question wording and response formats differ between cohorts owing to cultural and linguistic adaptations, both instruments capture the core construct of social and family engagement. These measurement differences are acknowledged as a limitation of cross-national comparison.

### Covariates

Covariates were selected based on their availability and comparability across both CHARLS and ELSA and their potential role as confounders in the association between stroke and cognitive decline. The following baseline covariates were included in fully adjusted models: age (continuous, centered at the cohort mean), sex (male/female), BMI (continuous, kg/m²), residence (urban/rural; CHARLS only, as ELSA does not have a comparable harmonized variable), educational attainment (harmonized three-level classification: below upper secondary, upper secondary and vocational training, tertiary), marital status (married or partnered versus other), smoking status (current smoker versus not), alcohol consumption (current drinker versus not), and comorbidity count (sum of six self-reported chronic conditions: hypertension, diabetes, heart disease, lung disease, cancer, and arthritis, ranging from 0 to 6). BMI for ELSA was derived from the nearest nurse visit. Since ELSA only collects measured height and weight at even-numbered waves, baseline BMI was obtained from Wave 2 (2004–2005, the most recent nurse visit before the Wave 3 baseline) when available; when Wave 2 BMI was missing or invalid, Wave 4 (2008–2009) BMI was used as a substitute.

### Statistical analysis

Baseline characteristics were summarized by social support level (high vs. low) using means and standard deviations for continuous variables and frequencies and percentages for categorical variables. Between-group differences were assessed using t-tests for continuous variables and chi-square tests for categorical variables, with standardized mean differences reported.

The association between incident stroke and longitudinal cognitive trajectories was evaluated using Linear Mixed-Effects Models (LMMs) with the lme4 and lmerTest packages in R. This approach directly models repeated cognitive measurements, accounts for within-person clustering through random effects, and estimates differential cognitive decline through interaction terms. The time scale was defined as years since baseline interview, centered at the cohort mean to reduce collinearity with interaction terms. Three linear mixed-effects models were estimated for each cohort. Model 1 included incident stroke, time, and their interaction, together with the full covariate set (age, sex, BMI, residence [CHARLS only], education, marital status, smoking, drinking, and comorbidity count), with a random intercept only. Model 2 retained the same fixed effects as Model 1 but added a random slope for time, allowing individual cognitive trajectories to vary in both baseline level and rate of decline; because this more flexible specification better captures known heterogeneity in individual decline trajectories, Model 2 was adopted as the primary, fully adjusted model reported in the Results and Table 3. Model 3 extended Model 2 by adding social support level (CHARLS) or family connection level (ELSA), together with its two-way and three-way interactions with incident stroke and time, to formally test moderation (Table 4). Full fixed-effect estimates for Models 1–3 are reported in Supplementary Tables S6/S10 (CHARLS) and S8/S12 (ELSA). In these models, incident stroke was defined as a time-invariant binary indicator (1 = participant reported stroke during any follow-up wave; 0 = never reported stroke during follow-up), social support/family connection was a binary indicator dichotomized at the sample median, and time was continuous years since baseline. The random effects structure (1 + time_years | ID) specified a random intercept and random slope for each participant, allowing individual baseline cognitive levels and rates of change to vary.

The primary coefficient of interest was the incident stroke × time interaction term, which estimates the differential annual cognitive decline (points per year) between incident stroke survivors and stroke-free participants, adjusting for all covariates. Negative values indicate faster cognitive decline in the stroke group. The incident stroke × social support/family connection × time three-way interaction directly tests whether social support or family connections moderate the association between stroke and cognitive decline; a positive coefficient indicates that higher social support attenuates stroke-related cognitive decline.

As a complementary approach, annualized cognitive change was estimated for each participant as the difference between the last available cognitive score and baseline cognitive score, divided by the corresponding time interval, and linear regression models were fit with the same sequential covariate adjustment. This provides an alternative, easily interpretable outcome specification.

Propensity score matching was performed as a sensitivity analysis to balance baseline covariates between incident stroke and stroke-free groups. Propensity scores were estimated using logistic regression with all baseline covariates (age, sex, BMI, education, marital status, smoking, drinking, comorbidity count, social support score, and residence for CHARLS). A 1:2 nearest-neighbor matching algorithm without replacement was applied using the MatchIt package. Covariate balance after matching was assessed using standardized mean differences, with values below 0.1 indicating adequate balance. The LMM analyses were then repeated on the PSM-matched samples.

Subgroup analyses were conducted by age (below 65 versus 65 years and above), sex (male versus female), and education level (below upper secondary, upper secondary, tertiary) to explore potential effect modification.

All analyses were performed using R version 4.3.3. Statistical significance was defined as a two-sided p-value below 0.05. Regression coefficients with 95% confidence intervals are reported as estimated annual changes in cognitive score (points per year) for the annualized change models, and as differential annual rates (points per year) for the LMM interaction terms. Sampling weights were not incorporated in our models, as the analytical focus was on estimating individual-level cognitive trajectories rather than generating nationally representative estimates.

## Results

### Baseline characteristics

After applying exclusion criteria, the final analytical sample comprised 11,569 CHARLS participants (mean age 59.1 ± 9.0 years; 52.0% female; mean baseline cognitive score 11.1 ± 3.9) and 5,713 ELSA participants (mean age 64.9 ± 9.7 years; 55.2% female; mean baseline cognitive score 14.5 ± 3.6) (Tables 1 and 2). Detailed stepwise exclusion counts are reported in Supplementary Table S1.

**Table 1:** Baseline characteristics of CHARLS participants by social support level

Characteristic	Low support (*n* = 8,440)	High support (*n* = 3,129)	*P*	SMD
Age, years, mean (SD)	56.84 (8.37)	62.60 (8.89)	< 0.001	0.667
Sex, n (%)			< 0.001	0.141
Male	4,152 (49.2)	1,320 (42.2)		
Female	4,288 (50.8)	1,809 (57.8)		
BMI, kg/m², mean (SD)	23.65 (3.82)	23.13 (3.93)	< 0.001	0.134
Education, n (%)			< 0.001	0.220
Below upper secondary	7,422 (87.9)	2,946 (94.2)		
Upper secondary	894 (10.6)	156 (5.0)		
Tertiary	124 (1.5)	27 (0.9)		
Marital status, n (%)			< 0.001	0.264
Not married	775 (9.2)	569 (18.2)		
Married	7,665 (90.8)	2,560 (81.8)		
Current smoker, n (%)			0.008	0.057
No	5,755 (68.2)	2,215 (70.8)		
Yes	2,685 (31.8)	914 (29.2)		
Current drinker, n (%)			0.135	0.032
No	5,113 (60.6)	1,944 (62.1)		
Yes	3,327 (39.4)	1,185 (37.9)		
Social support score (0–3), mean (SD)	0.86 (0.34)	2.05 (0.21)	< 0.001	4.141
Comorbidity count (0–6), mean (SD)	0.81 (0.92)	0.97 (0.97)	< 0.001	0.171
Residence, n (%)			< 0.001	0.113
Urban	3,174 (37.6)	1,008 (32.2)		
Rural	5,266 (62.4)	2,121 (67.8)		
Annualized cognitive change, mean (SD)†	−0.13 (0.91)	−0.27 (1.03)	< 0.001	0.147

*Abbreviations*: *BMI* Body mass index, *SD* Standard deviation, *SMD* Standardized mean difference. P values are from independent-samples t tests (continuous variables) or χ2 tests (categorical variables); SMD is the standardized mean difference between the low- and high-support groups. Social support score (range 0–3) is the sum of three binary indicators (weekly face-to-face contact with children, financial transfer from children/grandchildren, informal instrumental care from children/grandchildren); high support = score ≥ 2. † Annualized cognitive change is shown descriptively for context and was not used as a covariate in propensity-score matching

**Table 2:** Baseline characteristics of ELSA participants by family connection level

Characteristic	Low connection (*n* = 1,733)	High connection (*n* = 3,980)	*P*	SMD
Age, years, mean (SD)	65.27 (9.74)	64.52 (9.31)	0.006	0.078
Sex, n (%)			< 0.001	0.186
Male	881 (50.8)	1,656 (41.6)		
Female	852 (49.2)	2,324 (58.4)		
BMI, kg/m², mean (SD)	27.49 (4.82)	28.30 (5.14)	< 0.001	0.162
Education, n (%)			< 0.001	0.239
Below upper secondary	568 (32.8)	1,591 (40.0)		
Upper secondary	776 (44.8)	1,841 (46.3)		
Tertiary	389 (22.4)	548 (13.8)		
Marital status, n (%)			0.054	0.056
Not married	506 (29.2)	1,062 (26.7)		
Married	1,227 (70.8)	2,918 (73.3)		
Current smoker, n (%)			0.499	0.021
No	1,519 (87.7)	3,461 (87.0)		
Yes	214 (12.3)	519 (13.0)		
Current drinker, n (%)			0.022	0.069
No	148 (8.5)	420 (10.6)		
Yes	1,585 (91.5)	3,560 (89.4)		
Family connection score (0–4), mean (SD)	0.75 (0.44)	2.72 (0.75)	< 0.001	3.233
Comorbidity count (0–6), mean (SD)	1.04 (0.97)	1.11 (1.02)	0.016	0.070
Annualized cognitive change, mean (SD)†	−0.21 (0.67)	−0.17 (0.66)	0.022	0.066

*Abbreviations*: *BMI* Body mass index, *SD* Standard deviation, *SMD* Standardized mean difference. Family connection score (range 0–4) is the sum of four binary indicators of weekly contact (in person with children, by phone/email with children, in person with friends, in person with relatives), assessed at Wave 3 (baseline); high connection = score ≥ 2. Residence (urban/rural) is not reported for ELSA because a harmonized variable is not available in this cohort. † Shown descriptively; not used as a PSM-matching covariate

In CHARLS, the high social support group (*n* = 3,129, 27.0%) was older (62.6 vs. 56.8 years), more likely to be female (57.8% vs. 50.8%), had lower education levels, lower marriage rates (81.8% vs. 90.8%), and higher comorbidity burden (0.97 vs. 0.81 conditions) compared with the low support group (Table 1). In ELSA, the high family connection group $\:n=\mathrm{3,980,69.7}$ was slightly younger (64.5 vs. 65.3 years), more likely to be female (58.4% vs. 49.2%), had lower educational attainment, slightly higher marriage rates (73.3% vs. 70.8%), a slightly lower prevalence of current drinking (89.4% vs. 91.5%), and higher comorbidity burden (1.11 vs. 1.04 conditions) compared with the low connection group (Table 2).

During follow-up, 770 CHARLS participants (6.6%) and 347 ELSA participants (6.1%) reported incident stroke. The mean number of cognitive assessments per participant was 3.1 (range 2–4) in CHARLS and 5.6 (range 2–7) in ELSA. The mean annualized cognitive change across the full sample was − 0.16 ± 0.95 points per year in CHARLS and − 0.18 ± 0.66 points per year in ELSA.

### Incident stroke and cognitive decline

**Fig. 1:**
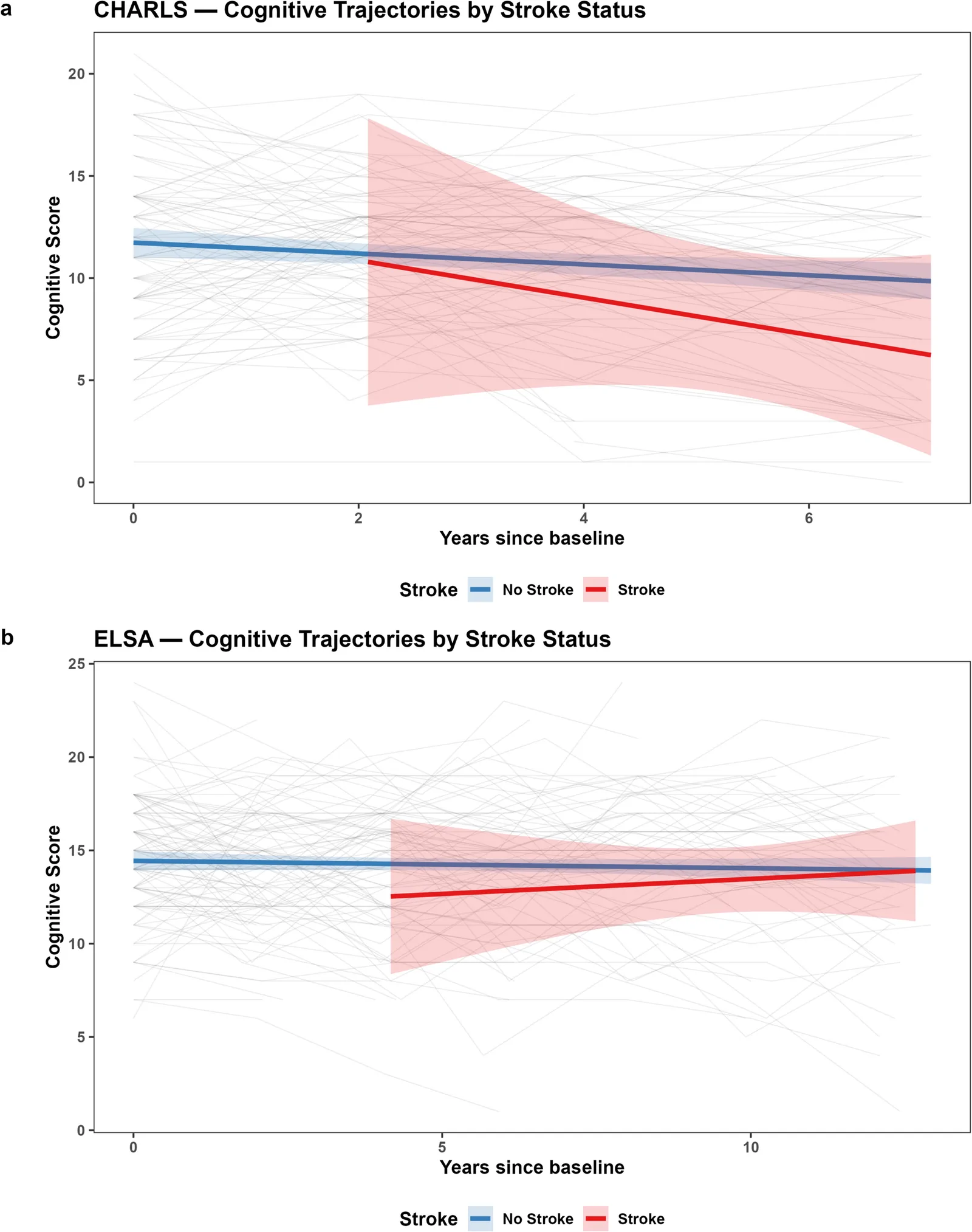
Cognitive trajectories by incident stroke status in CHARLS and ELSA. **a** CHARLS: individual cognitive trajectories (spaghetti plots) for a random sample of 100 participants, with linear smooth lines (LOESS) showing the overall trend for the incident stroke group (red) and stroke-free group (blue). Data from Waves 1–4 (2011–2018), with time measured in years since baseline (Wave 1). **b** ELSA: individual cognitive trajectories for a random sample of 100 participants, with linear smooth lines showing the overall trend for the incident stroke group (red) and stroke-free group (blue). Data from Waves 3–9 (2006–2019), with time measured in years since baseline (Wave 3). Cognitive score is the sum of orientation (0–4), immediate word recall (0–10), delayed word recall (0–10), and drawing test (0–1, CHARLS only), yielding a range of 0–25 (CHARLS) and 0–24 (ELSA). Shaded bands represent 95% confidence intervals. Abbreviations: CHARLS, China Health and Retirement Longitudinal Study; ELSA, English Longitudinal Study of Ageing

In CHARLS, incident stroke was associated with significantly faster cognitive decline in the linear mixed-effects models, consistent across all three model specifications (Table 3; Fig. 1a; Supplementary Table S6). In the fully adjusted model (Model 2), the stroke × time interaction was − 0.068 points per year (95% CI − 0.123 to − 0.013, *p* = 0.016), indicating that stroke survivors declined approximately 0.07 points per year faster than stroke-free participants; this association was also significant in the random-intercept-only model (Model 1: β = −0.066, *p* = 0.010) and remained consistent in the three-way interaction model (Model 3: β = −0.083, 95% CI − 0.148 to − 0.018, *p* = 0.013). In contrast, the annualized-change model — which collapses each participant’s repeated measurements into a single per-person slope and therefore has less statistical power than the LMM — showed a directionally consistent but non-significant association (β = −0.023 points per year, *p* = 0.560; Supplementary Table S5).

In ELSA, incident stroke was robustly associated with faster cognitive decline (Table 3; Fig. 1b). In the fully adjusted linear mixed-effects model, the stroke × time interaction was − 0.190 points per year (95% CI − 0.229, − 0.152, *p* < 0.001), indicating that stroke survivors declined approximately 0.19 points per year faster than stroke-free participants. This association was consistent in the three-way interaction model (stroke × time interaction: −0.260 points per year, 95% CI − 0.335 to − 0.184, *p* < 0.001). The annualized change models also showed significant effects, with the fully adjusted model yielding a stroke main effect of − 0.146 points per year (95% CI − 0.220 to − 0.071, *p* < 0.001; Supplementary Table S7); full LMER coefficients are reported in Supplementary Table S8.

**Table 3:** Association between incident stroke and estimated annual cognitive decline (linear mixed-effects models, Model 2: fully adjusted)

Sample	Cohort	*N* (stroke/control)	β (points/year)	95% CI	*P*
Full sample	CHARLS	770 / 10,799	−0.068	−0.123, − 0.013	0.016
Full sample	ELSA	347 / 5,366	−0.190	−0.229, − 0.152	< 0.001
PSM-matched	CHARLS	769 / 1,538	−0.029	−0.095, 0.037	0.386
PSM-matched	ELSA	306 / 612	−0.150	−0.200, − 0.100	< 0.001

β is the incident-stroke × time interaction coefficient (points per year) from Model 2 (adjusts for age, sex, BMI, residence [CHARLS only], education, marital status, smoking, drinking, comorbidity count; random intercept and slope for each participant). A negative β indicates faster annual cognitive decline in the incident-stroke group than the stroke-free group. PSM = 1:2 nearest-neighbor propensity-score-matched sample. 95% CIs were recalculated as β ± 1.96×SE from the model output

The consistency in the direction and significance of these associations supports the robustness of accelerated cognitive decline following incident stroke across both cohorts.

### Moderation by social support and family connections

**Fig. 2:**
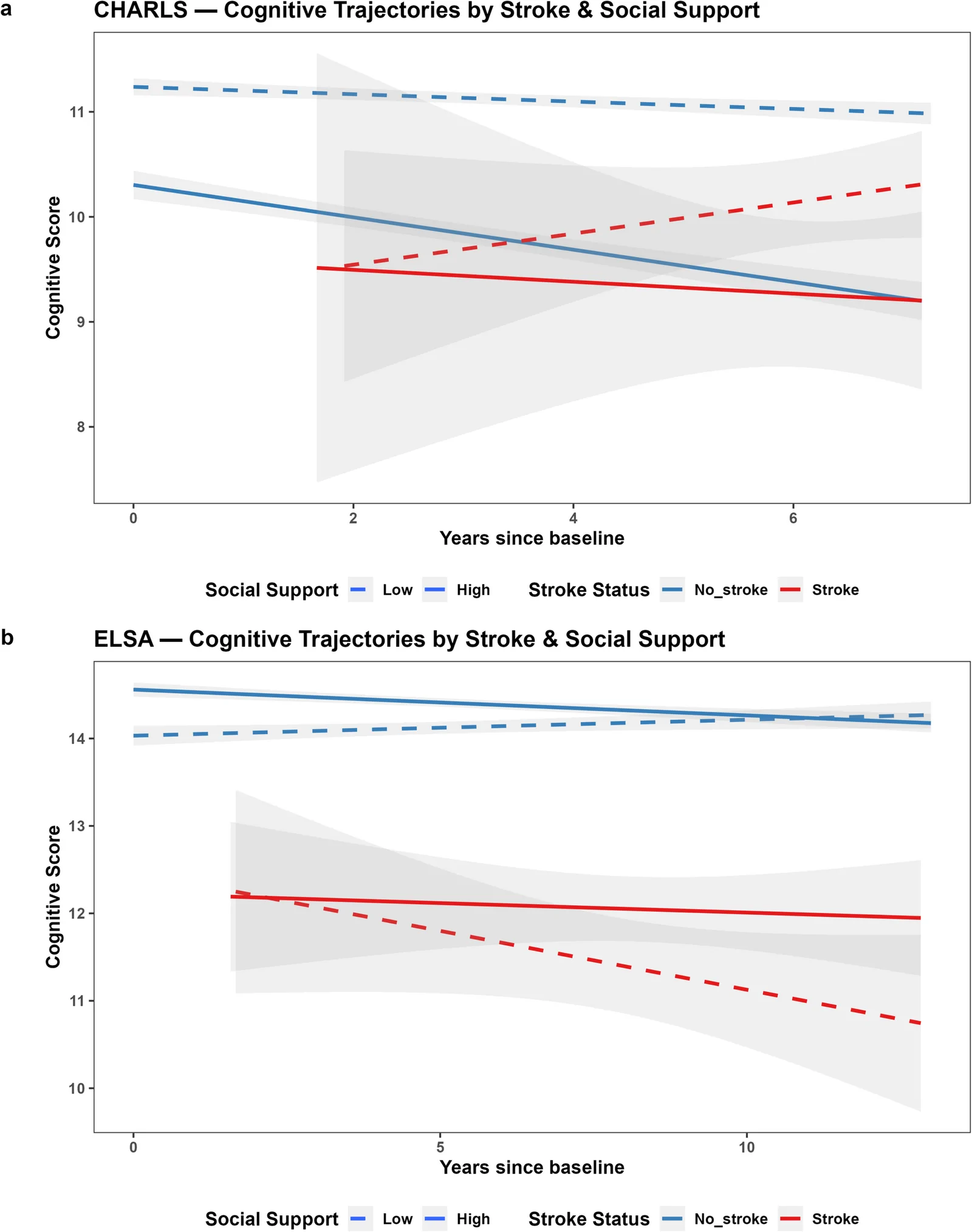
Cognitive trajectories stratified by stroke status and social support or family connection level. **a** CHARLS: trajectories for four groups defined by the combination of incident stroke status (stroke vs. no stroke) and social support level (high vs. low, median split of the 0–3 composite score). High social support was defined as having at least two of the following: weekly face-to-face contact with children, financial transfers from children, and informal care from children. **b** ELSA: trajectories for four groups defined by incident stroke status and family connection level (high vs. low, median split of the 0–4 composite score). High family connection was defined as having at least two of the following: weekly in-person contact with children, weekly phone/email contact with children, weekly in-person contact with friends, and weekly in-person contact with relatives. Lines represent linear model fits with 95% confidence bands

In CHARLS, the three-way interaction between incident stroke, social support, and time did not reach statistical significance in the full sample analysis (β = +0.082, 95% CI − 0.037 to + 0.202, *p* = 0.177; Table 4; Fig. 2a). In the PSM-matched sensitivity analysis (769 stroke versus 1,538 control participants), the three-way interaction approached significance (β = +0.121, 95% CI − 0.020 to + 0.262, *p* = 0.096; Table 4). The coefficient carries a positive sign, tentatively consistent with a potential buffering effect of social support on stroke-related cognitive decline, though the association fell short of conventional statistical significance in the full sample.

**Table 4:**
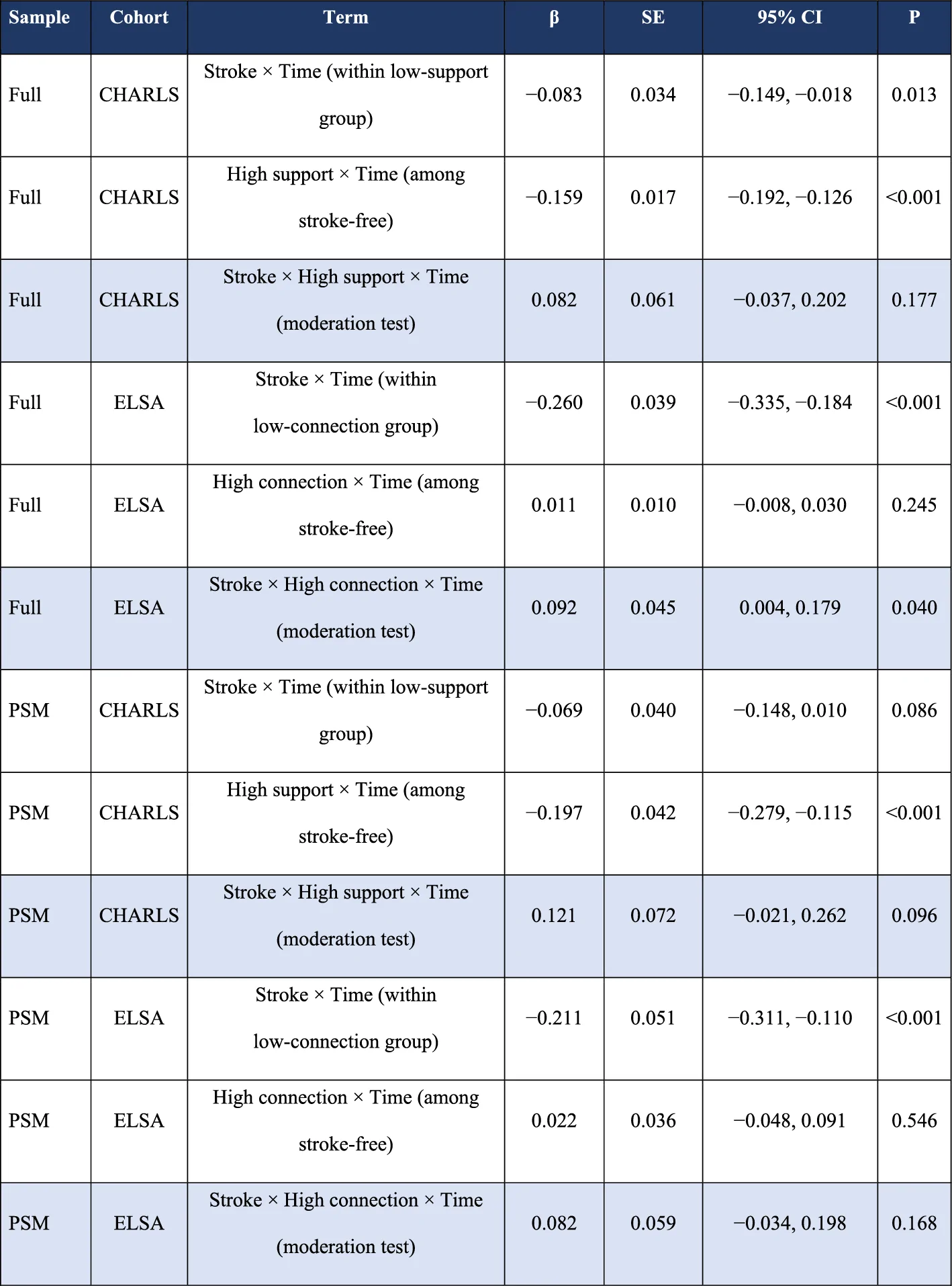
Formal test of moderation: three-way interaction among incident stroke, social support/family connection level, and time (Model 3)

All estimates are from Model 3 (Model 2 covariates plus social support/family connection level and its interactions with stroke and time; random intercept and slope). “Stroke × Time” is the simple effect of incident stroke on annual cognitive change specifically within the low support/connection (reference) group — it is NOT the marginal/average stroke effect reported in Table 3. “High support/connection × Time” is the simple effect of support/connection level among stroke-free participants. The three-way interaction (shaded rows) is the formal statistical test of moderation: it tests whether the stroke-associated difference in annual cognitive decline itself differs between high and low support/connection groups; a positive coefficient indicates attenuation (buffering) of stroke-related decline in the high support/connection group. 95%CIs recalculated as β ± 1.96×SE

In ELSA, family connections significantly moderated stroke-related cognitive decline in the full sample (Table 4; Fig. 2b). The three-way interaction was statistically significant(β = +0.092, 95% CI 0.004 to 0.179, *p* = 0.040), indicating that among participants with high family connections, the acceleration of cognitive decline following stroke was attenuated by approximately 0.09 points per year compared with those with low family connections. In the PSM-matched analysis (306 stroke versus 612 control participants), the three-way interaction remained in the same direction but did not reach significance (β = +0.082, *p* = 0.168), likely reflecting reduced statistical power in the smaller matched sample.

### Propensity score matching

**Fig. 3:**
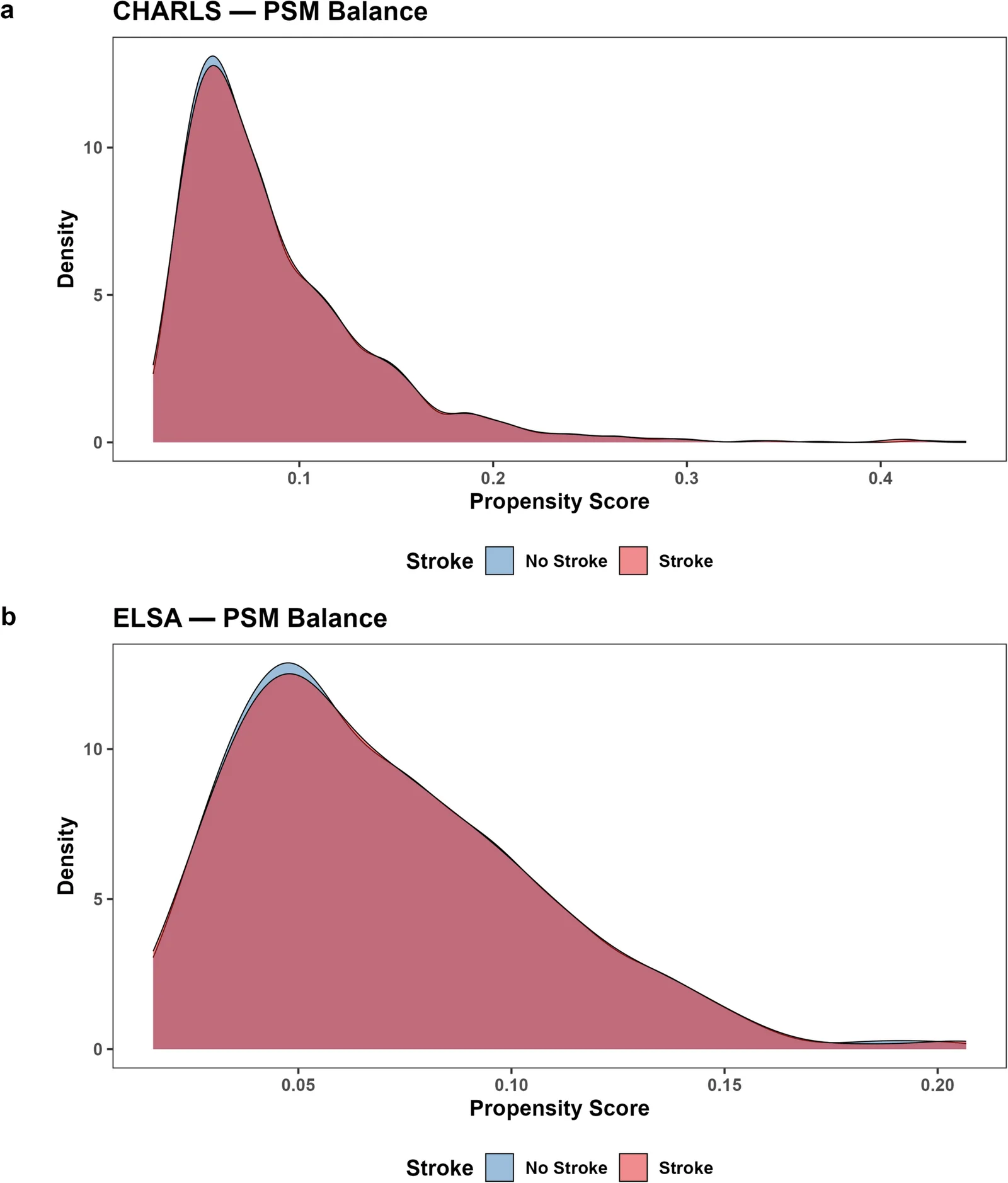
Propensity score density distributions before and after 1:2 nearest-neighbor matching. **a** CHARLS: density of estimated propensity scores for incident stroke (red) and stroke-free (blue) groups. Propensity scores were estimated from a logistic regression model including age, sex, BMI, residence, education, marital status, smoking, drinking, comorbidity count, and social support score. After matching (769 stroke survivors matched to 1,538 controls), all standardized mean differences were below 0.1. **b** ELSA: same as (**a**) for the ELSA cohort (306 stroke survivors matched to 612 controls). Covariates included age, sex, BMI, education, marital status, smoking, drinking, comorbidity count, and family connection score (residence not available in ELSA). Abbreviations: PSM, propensity score matching; SMD, standardized mean difference; BMI, body mass index

**Fig. 4:**
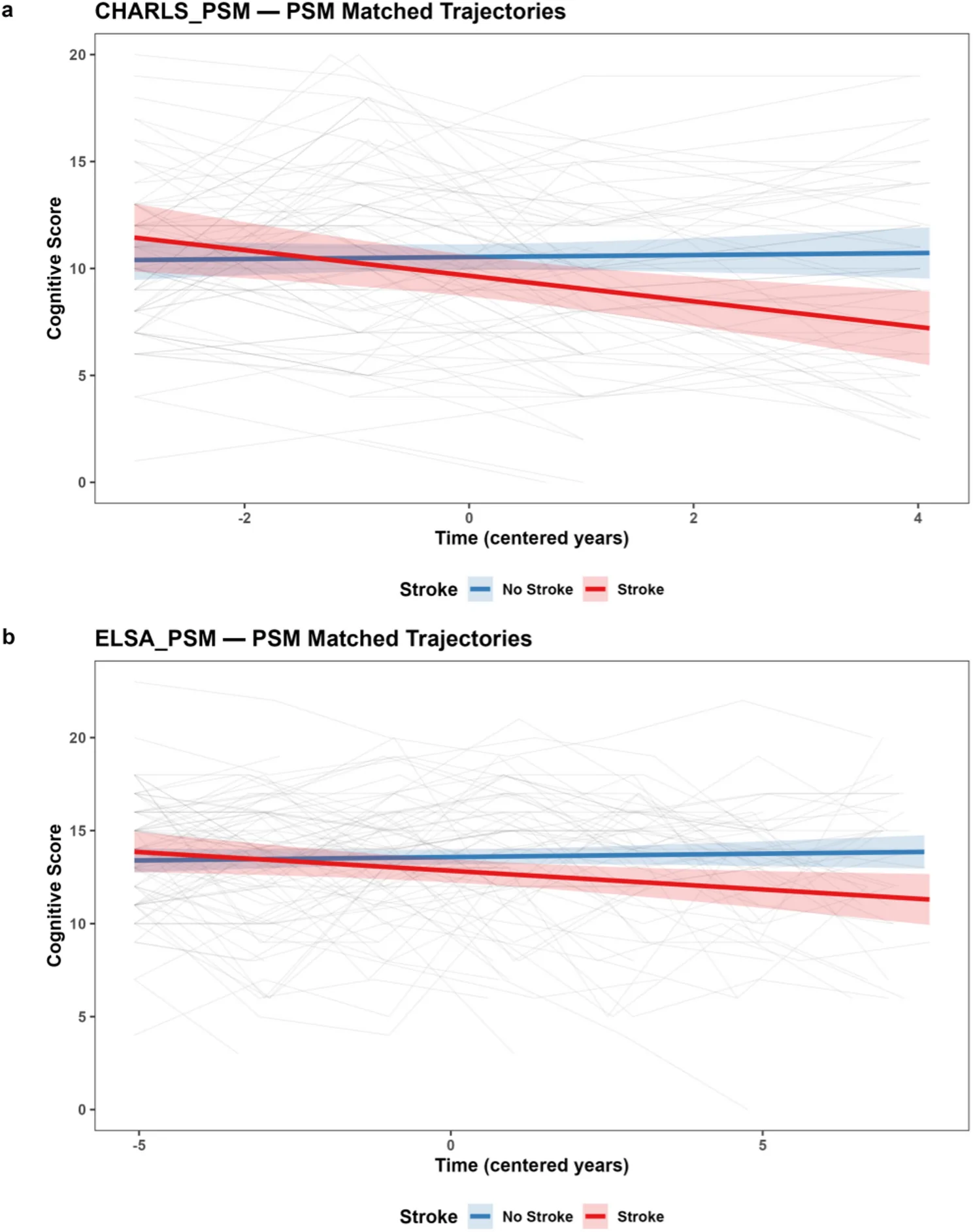
PSM-matched cognitive trajectories by incident stroke status. **a** CHARLS: cognitive trajectories in the 1:2 PSM-matched sample (*N* = 2,307; 769 stroke, 1,538 control). Linear smooth lines with 95% confidence bands show the estimated cognitive trajectories for each group. All baseline covariates were balanced after matching (all SMD < 0.1). **b** ELSA: cognitive trajectories in the 1:2 PSM-matched sample (*N* = 918; 306 stroke, 612 control). The larger separation between groups in ELSA compared with CHARLS may reflect the longer follow-up period (13.0 vs. 7.2 years) and older baseline age

After 1:2 propensity score matching, all baseline covariates were well-balanced between incident stroke and stroke-free groups, with all standardized mean differences below 0.1 (Fig. 3a and b; Supplementary Tables S3 and S4). In the matched CHARLS sample of 2,307 participants, the stroke × time interaction was attenuated and no longer statistically significant (− 0.029 points per year, 95% CI − 0.095 to 0.037, *p* = 0.386; Table 3; Fig. 4a) (full coefficients in Supplementary Table S10). In the matched ELSA sample of 918 participants, the association remained robust (stroke × time interaction: −0.150 points per year, 95% CI − 0.200 to − 0.100, *p* < 0.001; Table 3; Fig. 4b) (full coefficients in Supplementary Table S12). The PSM results were consistent with the full-sample findings for ELSA, whereas for CHARLS the stroke-associated decline was attenuated and no longer significant after matching, indicating that the full-sample CHARLS association should be interpreted with some caution.

### Subgroup analyses

Subgroup analyses by age, sex, and education were generally consistent with the main findings, though confidence intervals were wide in some strata due to reduced sample sizes (Table 5). In ELSA, the stroke × time interaction was significant across all subgroups, with somewhat larger effect estimates among females (β = −0.181, *p* < 0.001) and those with Tertiary (β = −0.202, *p* < 0.001). In CHARLS, the stroke × time interaction was directionally consistent across subgroups but reached statistical significance only among male participants (β = −0.077, *p* = 0.026) and in the below-upper-secondary education stratum (β = −0.057, *p* = 0.037), which represented the majority (89.6%) of the CHARLS sample.

**Table 5:** Subgroup analyses of the association between incident stroke and estimated annual cognitive decline (linear mixed-effects models, Model 2: fully adjusted), by age, sex, and education

Sample	Cohort	Subgroup	Level	*N* (stroke/control)	β (points/year)	95% CI	*P*
Full sample	CHARLS	Age	< 65 years	532 / 8,188	-0.047	-0.105, 0.010	0.107
Full sample	CHARLS	Age	≥ 65 years	227 / 2,474	-0.063	-0.164, 0.039	0.226
Full sample	CHARLS	Sex	Male	376 / 5,061	-0.077	-0.145, -0.009	0.026
Full sample	CHARLS	Sex	Female	383 / 5,601	-0.052	-0.126, 0.022	0.171
Full sample	CHARLS	Education	Below upper secondary	692 / 9,528	-0.057	-0.111, -0.003	0.037
Full sample	CHARLS	Education	Upper secondary	61 / 989	-0.075	-0.226, 0.075	0.324
Full sample	CHARLS	Education	Tertiary	6 / 145	-0.395	-0.844, 0.054	0.086
Full sample	ELSA	Age	< 65 years	96 / 2,997	-0.121	-0.167, -0.074	< 0.001
Full sample	ELSA	Age	≥ 65 years	210 / 2,410	-0.159	-0.202, -0.116	< 0.001
Full sample	ELSA	Sex	Male	156 / 2,381	-0.174	-0.218, -0.131	< 0.001
Full sample	ELSA	Sex	Female	150 / 3,026	-0.181	-0.226, -0.137	< 0.001
Full sample	ELSA	Education	Below upper secondary	139 / 2,020	-0.175	-0.225, -0.125	< 0.001
Full sample	ELSA	Education	Upper secondary	128 / 2,489	-0.161	-0.207, -0.116	< 0.001
Full sample	ELSA	Education	Tertiary	39 / 898	-0.202	-0.286, -0.118	< 0.001
PSM-matched	CHARLS	Age	< 65 years	532 / 1,037	-0.035	-0.105, 0.035	0.328
PSM-matched	CHARLS	Age	≥ 65 years	227 / 471	-0.003	-0.121, 0.114	0.956
PSM-matched	CHARLS	Sex	Male	376 / 747	-0.050	-0.131, 0.031	0.223
PSM-matched	CHARLS	Sex	Female	383 / 761	0.004	-0.086, 0.094	0.933
PSM-matched	CHARLS	Education	Below upper secondary	692 / 1,373	-0.002	-0.066, 0.062	0.955
PSM-matched	CHARLS	Education	Upper secondary	61 / 126	-0.183	-0.371, 0.004	0.056
PSM-matched	ELSA	Age	< 65 years	96 / 217	-0.104	-0.164, -0.044	< 0.001
PSM-matched	ELSA	Age	≥ 65 years	210 / 395	-0.156	-0.211, -0.101	< 0.001
PSM-matched	ELSA	Sex	Male	156 / 310	-0.135	-0.191, -0.079	< 0.001
PSM-matched	ELSA	Sex	Female	150 / 302	-0.144	-0.203, -0.085	< 0.001
PSM-matched	ELSA	Education	Below upper secondary	139 / 301	-0.142	-0.207, -0.078	< 0.001
PSM-matched	ELSA	Education	Upper secondary	128 / 245	-0.139	-0.199, -0.079	< 0.001
PSM-matched	ELSA	Education	Tertiary	39 / 66	-0.136	-0.245, -0.028	0.014

β is the incident-stroke × time interaction coefficient (points per year) from Model 2 (adjusts for age, sex, BMI, residence [CHARLS only], education, marital status, smoking, drinking, comorbidity count, as applicable; random intercept and slope for each participant), estimated separately within each subgroup stratum. A negative β indicates faster annual cognitive decline in the incident-stroke group than the stroke-free group within that stratum. These are stratified point estimates, not formal statistical tests of effect modification; no subgroup × stroke × time interaction term was fitted, so apparent differences between strata (e.g., by sex or education) should not be interpreted as statistically confirmed effect modification. N (stroke/control) gives the number of incident-stroke and stroke-free participants within each stratum. PSM = 1:2 nearest-neighbor propensity-score-matched sample (see Table 3). The tertiary-education stratum is not reported for the CHARLS PSM-matched sample because of an insufficient number of stroke cases in that cell. 95% CIs were recalculated as β ± 1.96×SE from the model output. Age was dichotomized at 65 years; education follows the harmonized three-level classification described in Methods

### Annual cognitive change

**Fig. 5:**
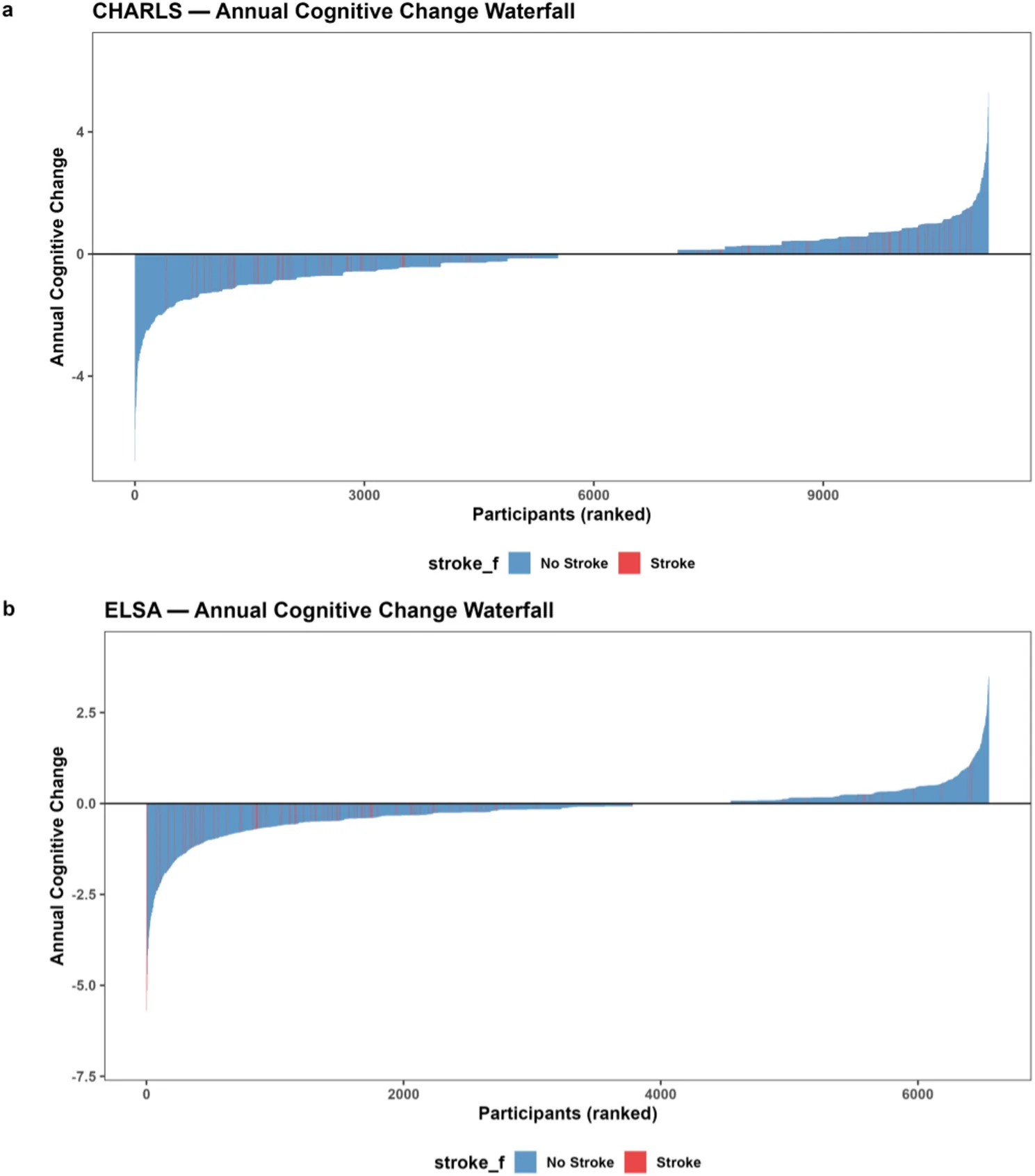
Waterfall plot of individual annual cognitive change rates. **a** CHARLS: each vertical bar represents one participant *N*=11,569, ranked from the most negative (fastest decline) to the most positive (greatest improvement) annual change rate. Bars are colored by incident stroke status (red = stroke during follow-up, blue = no stroke). The horizontal dashed line at zero indicates no change. Annual change was calculated as (final cognitive score − baseline cognitive score) / follow-up years. **b** ELSA: waterfall plot for the ELSA cohort *N*=5,713

Annualized cognitive change scores, calculated as the difference between the last available and baseline cognitive assessments divided by follow-up time, provided a complementary perspective to the longitudinal models (Fig. 5). Across the full sample, the mean annual cognitive decline was − 0.16 ± 0.95 points per year in CHARLS and − 0.18 ± 0.66 points per year in ELSA. In CHARLS, incident stroke survivors showed numerically greater annual decline compared with stroke-free participants, though the difference was not statistically significant in annualized change models after full covariate adjustment (Supplementary Tables S5 and S9). In ELSA, incident stroke was significantly associated with greater annual decline in both the full sample and PSM-matched analyses (Supplementary Tables S7 and S11).

### Cross-cohort synthesis

Across both cohorts, incident stroke was consistently associated with accelerated cognitive decline. The estimated annual cognitive decline was approximately 0.07 to 0.08 points per year faster in CHARLS stroke survivors and 0.19 to 0.26 points per year faster in ELSA stroke survivors compared with their stroke-free counterparts, based on the longitudinal mixed-effects models. The apparently larger effect size in ELSA may reflect the longer follow-up period (13.0 versus 7.2 years), cohort differences in age structure (ELSA participants were approximately six years older at baseline), and differences in cognitive reserve and healthcare access between the two populations. Regarding social moderation, family connections significantly attenuated stroke-related cognitive decline in the ELSA cohort (three-way interaction *p* = 0.040), while social support showed a directionally consistent but non-significant pattern in CHARLS * p=0.177*. It should be noted that the social support construct differed between cohorts, with intergenerational support being assessed in CHARLS and broader family and friend contact being assessed in ELSA, which may contribute to the observed difference in statistical significance. These findings should be interpreted as qualitatively consistent patterns across two culturally distinct populations rather than as a formal quantitative comparison of effect magnitudes.

## Discussion

This cross-national study shows that stroke was associated with significantly faster cognitive decline in older adults, while higher social support and stronger family connections were associated with slower cognitive decline among stroke survivors. Our findings extend previous research by providing evidence from two culturally distinct longitudinal cohorts and explicitly examining subgroup associations of psychosocial factors with post-stroke cognitive decline.

### Stroke and accelerated cognitive decline

Our results are consistent with cognitive decline rates being significantly higher among stroke patients than stroke-free individuals, aligning with contemporary epidemiological evidence. Post-stroke cognitive impairment represents a major source of morbidity and mortality, affecting 30% to over 70% of stroke survivors depending on assessment methods and populations [6–10]. The mechanisms underlying accelerated post-stroke cognitive decline are multifactorial. Stroke location and severity critically determine specific cognitive deficits [8, 13], while vascular risk factors including hypertension, diabetes, and white matter changes contribute to progressive deterioration through cerebral hypoperfusion, ongoing white matter damage, and neuroinflammation [8, 15, 37]. Recent evidence emphasizes roles of baseline cognitive function, stroke severity, depression, and metabolic factors in predicting PSCI development [10, 38].

Cognitive trajectories show substantial heterogeneity among stroke survivors. While some demonstrate improvement, particularly within three months post-stroke [20], others experience persistent or progressive decline culminating in dementia. Longitudinal research tracking cognitive function pre- and post-stroke suggests cerebrovascular disease processes begin years before clinical events, with decline evident across this continuum [17, 18]. This temporal pattern underscores the importance of identifying factors associated with cognitive trajectories throughout stroke recovery, which may warrant further investigation as potential intervention targets.

### Associations of social support and family connections with cognitive decline

This study’s primary innovation lies in comparative analysis of stroke’s impact on cognitive decline across Chinese and UK elderly populations, addressing a significant gap in cross-national research on psychosocial factors related to cognitive outcomes. Our findings show that social support and family connections were associated with better post-stroke cognitive function, extending previous literature emphasizing social network importance in cognitive health [39, 40].

Protective mechanisms of social support are well-established in aging literature. Functional social support, particularly emotional and instrumental support, consistently associates with higher cognitive function in middle-aged and older adults [22, 41]. Meta-analysis of 34 studies demonstrated poor social relationships significantly associated with cognitive decline, with odds ratios of 1.11 to 1.15 [25]. Social support operates through multiple pathways: reducing stress and negative emotions [42], promoting healthy behaviors [43], enhancing self-worth and motivation [44], and potentially reducing inflammatory responses [23].

In stroke recovery contexts, family connections represent particularly potent social engagement forms. Research consistently shows family support positively impacts functional recovery, with particularly strong effects in severely impaired patients [26]. Recent interventions demonstrated family-based programs improve family function, decrease caregiver burden, reduce depression, and enhance functional status in stroke survivors [28]. Family involvement in rehabilitation helps caregivers feel prepared and improves patient outcomes [29, 30].

Our results indicate that among stroke survivors, higher family connections in ELSA were associated with a statistically significant attenuation of stroke-related cognitive decline, and a similar directional pattern was observed for social support in CHARLS, although the CHARLS association did not reach statistical significance. Although our analysis formally tests statistical moderation via the three-way interaction, the non-randomized nature of social support/family connection means causal moderation mechanisms cannot be established [45, 46].

### Clinical implications and intervention opportunities

These findings suggest that social support and family connections may be worth considering in stroke rehabilitation. Prior intervention research indicates that psychosocial support can improve recovery outcomes and quality of life for stroke survivors [29, 30, 47], and our associational findings are broadly consistent with this literature.

Several practical implications emerge for clinical practice. First, routine assessment of social support and family function should integrate into comprehensive stroke evaluations, as these factors predict cognitive trajectories and rehabilitation outcomes [26]. Second, healthcare systems should implement family-centered care models actively involving family members in rehabilitation planning and delivery [29, 30]. Recent evidence demonstrates family-based interventions effectively improve patient outcomes while reducing caregiver burden [28].

Third, interventions targeting social engagement should be developed specifically for stroke populations. While community-based programs promoting social participation show promise for preventing cognitive decline in general older populations [48, 49], tailored programs addressing unique needs and barriers faced by stroke survivors are needed. Virtual platforms may overcome mobility and transportation barriers, with studies showing virtual family conferences effectively facilitate education and transition planning in stroke rehabilitation [30].

These findings may influence health policy by advocating for integrated support systems prioritizing mental health and social engagement in rehabilitation programs [50, 51]. Given stroke’s substantial economic burden, investing in psychosocial interventions represents a potentially cost-effective strategy for improving long-term outcomes. Healthcare systems should consider reimbursement policies supporting family caregiver training, respite care, and community-based social engagement programs for stroke survivors.

### Study limitations and future research directions

Several limitations warrant acknowledgment. First, reliance on self-reported stroke status and social support introduces potential reporting bias [52]. Future research should incorporate objective assessments, including neuroimaging confirmation and validated observational measures of social interactions.

Second, incident stroke was modeled as a time-invariant exposure: participants who experienced a stroke at any point during follow-up were classified as the incident-stroke group for their entire observation period, rather than switching status at the specific wave in which the stroke occurred. Consequently, the estimated stroke × time interaction reflects the average difference in decline slope between ever-stroke and never-stroke participants across the full follow-up window, which necessarily includes each stroke survivor’s pre-stroke observations; this could attenuate the true magnitude of post-stroke acceleration in decline and cannot fully exclude reverse causation, whereby participants already on a steeper pre-stroke trajectory may be more likely to subsequently experience a stroke. A time-varying specification that switches stroke status at the wave of onset would provide a more temporally precise test and should be considered in future work with more frequent stroke-onset ascertainment.

Third, while we adjusted for numerous covariates, residual confounding from unmeasured factors—including genetic predisposition, pre-stroke cognitive reserve, stroke subtypes and locations, and acute care quality—cannot be excluded [15, 53]. The observational design precludes definitive causal inferences about social support’s protective associations. In addition, although economic factors such as income or household wealth may also be relevant confounders, these variables were not included in the primary models because of limited harmonization and comparability across the two cohorts.

Fourth, selection bias may have threatened the internal validity of our findings through both differential loss to follow-up and exclusion due to missing data. Although both cohorts are nationally based, participants with more severe stroke, poorer health status, lower educational attainment, or greater cognitive impairment may have been less likely to remain in the study over time or to provide complete sociodemographic and health-related information. Consequently, the final analytical sample may have represented a relatively healthier and less vulnerable subset of participants. This may have resulted in underestimation of the true rate of post-stroke cognitive decline and may also have attenuated the observed associations of social support and family connections with cognitive decline [54].

Fifth, binary categorization of social support and family connections, while facilitating interpretation, may obscure nuanced dose-response relationships and fails to capture social relationship complexity [25, 55]. Future studies should examine specific support components (emotional, instrumental, informational) and their differential effects on cognitive outcomes.

Sixth, we did not assess social relationship quality or potential negative aspects, such as family conflict or caregiver strain, which may also influence cognitive outcomes [31, 56]. Research examining both positive and negative social relationship dimensions would provide more complete understanding of psychosocial influences on post-stroke cognition.

Future research should address these limitations through multi-center studies with larger, more diverse samples to enhance generalizability and examine effect modification by demographic and clinical characteristics [57]. Incorporating objective social interaction measures through digital monitoring or ecological momentary assessment could reduce self-report bias. Longer follow-up beyond two years would enable better assessment of long-term cognitive trajectories and sustainability of social support effects [58].

Critically, intervention trials are needed to establish causality and identify effective strategies for enhancing psychosocial factors to improve cognitive health in stroke patients [59, 60]. Such trials should test specific, theory-driven interventions targeting social support networks, family caregiver training, and community-based social engagement programs. Comparative effectiveness research examining different intervention modalities would inform optimal implementation strategies. Research exploring biological mechanisms linking social support to cognitive outcomes—through stress biomarkers, inflammatory markers, or neuroimaging measures of brain structure and function—would enhance understanding of pathways through which psychosocial factors exert protective effects [61, 62].

Finally, we conducted all analyses separately within the CHARLS and ELSA cohorts rather than using a pooled dataset. Therefore, we did not perform a formal statistical test to directly compare the magnitude of the associations across China and England. In addition, although the cognitive and social variables were selected to capture broadly comparable constructs, differences in question wording, response formats, and cultural interpretations of social support and family connection may have affected measurement comparability between the two cohorts. Accordingly, our cross-cohort interpretation should be understood as qualitative consistency in the observed patterns rather than a formal quantitative comparison of effect sizes. Future studies using more fully harmonized cross-national measures are needed to better assess potential heterogeneity in these associations.

## Conclusion

This study demonstrates that stroke was significantly associated with faster cognitive decline in older adults across two culturally distinct populations. Among stroke survivors, higher family connections were associated with a statistically significant attenuation of this decline in ELSA, while a similar but non-significant directional pattern was observed for social support in CHARLS. Cognitive impairment prevalence among stroke survivors ranges from 15% to 70% depending on assessment methods [11, 12]. Our findings extend this knowledge by quantifying stroke-associated cognitive decline magnitude and identifying modifiable psychosocial factors associated with slower decline.

The broadly consistent pattern of associations across the Chinese and English cohorts suggests that social and family engagement may be relevant to post-stroke cognitive outcomes across different cultural contexts, although formal cross-cohort statistical comparison was not performed and causal or universal mechanisms cannot be inferred from this observational design. This cross-national evidence may inform future consideration of integrating social support assessment and enhancement into routine stroke rehabilitation practices worldwide.

Significant questions remain regarding optimal strategies for translating these epidemiological findings into effective interventions. Future research should prioritize multi-center randomized controlled trials to validate these findings and test interventions aimed at strengthening social support networks among stroke survivors [57, 63]. Research should examine the mechanisms underlying the association between social support and cognitive outcomes, optimal timing and intensity of psychosocial interventions, and effective integration into existing stroke rehabilitation frameworks. Understanding treatment response heterogeneity and identifying subgroups most likely to benefit will be crucial for developing personalized rehabilitation approaches.

This study provides evidence that while stroke is associated with worse cognitive health in older adults, this association may be attenuated among individuals with strong social support and family connections. These findings support viewing stroke recovery not solely through a biomedical lens, but as a biopsychosocial process in which social relationships may play a meaningful role. Future intervention research is needed to determine whether family-centered, socially engaged models of stroke rehabilitation can improve cognitive outcomes and quality of life for the growing population of stroke survivors worldwide.

## Supplementary Information

Supplementary Material 1.

## Data Availability

The datasets supporting the conclusions of this article are available publicly.

## Abbreviations

CHARLS: China Health and Retirement Longitudinal Study
ELSA: English Longitudinal Study of Ageing
PSCI: Post-stroke cognitive impairment
PSM: Propensity score matching
