# Supplementary material for: Stroke and cognitive decline: associations of social support and family connections in CHARLS and ELSA cohorts
Source: BMC Public Health. 2026 Aug 6;26:3182. doi: 10.1186/s12889-026-28890-0 (PMC13729641; doi:10.1186/s12889-026-28890-0)
Supplement: Supplementary file 1 [file 12889_2026_28890_MOESM1_ESM.docx]

Stroke and Cognitive Decline: Associations of Social Support and Family Connections in CHARLS and ELSA Cohorts

## 1.1. Contents

Table S1. CHARLS — Stepwise exclusion of participants.

Table S2. ELSA — Stepwise exclusion of participants.

Table S3. CHARLS — PSM-matched baseline characteristics by incident stroke status.

Table S4. ELSA — PSM-matched baseline characteristics by incident stroke status.

Table S5. CHARLS — Full sample annualized change model coefficients.

Table S6. CHARLS — Full sample linear mixed-effects model coefficients.

Table S7. ELSA — Full sample annualized change model coefficients.

Table S8. ELSA — Full sample linear mixed-effects model coefficients.

Table S9. CHARLS — PSM-matched annualized change model coefficients.

Table S10. CHARLS — PSM-matched linear mixed-effects model coefficients.

Table S11. ELSA — PSM-matched annualized change model coefficients.

Table S12. ELSA — PSM-matched linear mixed-effects model coefficients.

Figure S1. CHARLS — Individual cognitive trajectories by stroke status.

Figure S2. ELSA — Individual cognitive trajectories by stroke status.

Figure S3. CHARLS — Cognitive trajectories by stroke and social support.

Figure S4. ELSA — Cognitive trajectories by stroke and family connection.

Figure S5. CHARLS — Annual cognitive change waterfall plot.

Figure S6. ELSA — Annual cognitive change waterfall plot.

Figure S7. CHARLS — Propensity score density plot.

Figure S8. ELSA — Propensity score density plot.

Figure S9. CHARLS — PSM-matched cognitive trajectories.

Figure S10. ELSA — PSM-matched cognitive trajectories.

Table S1. CHARLS — Stepwise exclusion of participants.

| **Step** | **N** | **Excluded** | **Reason** |
| --- | --- | --- | --- |
| 1_Raw | 25586 | 0 | CHARLS W1 initial |
| 2_Age<45 | 16931 | 8655 | Age<45 |
| 3_Base_stroke | 16462 | 469 | Prevalent stroke |
| 4_BMI_range | 12754 | 3708 | BMI outlier |
| 5_No_followup | 11606 | 1148 | No follow-up cog |
| 6_Complete_Case | 11569 | 37 | Missing covariates |
| *Abbreviations: BMI, body mass index; W1-W4, Waves 1 to 4.* | | | |

Table S2. ELSA — Stepwise exclusion of participants.

| **Step** | **N** | **Excluded** | **Reason** |
| --- | --- | --- | --- |
| 1_Raw | 19802 | 0 | ELSA W3 initial |
| 2_Age<50 | 9338 | 10464 | Age<50 |
| 3_Base_stroke | 8903 | 435 | Prevalent stroke |
| 4_BMI_missing | 7274 | 1629 | Missing BMI |
| 5_No_followup | 6554 | 720 | No follow-up cog |
| 6_Complete_Case | 5713 | 841 | Missing covariates |
| *BMI for ELSA Wave 3 baseline was derived from Wave 2 (or Wave 4) nurse visit measurements.* | | | |

Table S3. CHARLS — PSM-matched baseline characteristics by incident stroke status.

| **V1** | **level** | **No_Stroke** | **Stroke** | **p** | **test** | **SMD** |
| --- | --- | --- | --- | --- | --- | --- |
| n |  | 1538 | 769 |  |  |  |
| age..mean..SD.. |  | 60.65 (9.01) | 60.51 (8.31) | 0.720 |  | 0.016 |
| sex.... | Male | 756 (49.2) | 376 (48.9) | 0.941 |  | 0.005 |
| X | Female | 782 (50.8) | 393 (51.1) |  |  |  |
| bmi..mean..SD.. |  | 24.52 (4.23) | 24.47 (4.06) | 0.794 |  | 0.012 |
| education.... | 1_Below_upper_secondary | 1403 (91.2) | 702 (91.3) | 0.842 |  | 0.025 |
| X.1 | 2_Upper_secondary | 126 (8.2) | 61 (7.9) |  |  |  |
| X.2 | 3_Tertiary | 9 (0.6) | 6 (0.8) |  |  |  |
| marital.... | Not_married | 210 (13.7) | 112 (14.6) | 0.595 |  | 0.026 |
| X.3 | Married | 1328 (86.3) | 657 (85.4) |  |  |  |
| smoke.... | No | 1053 (68.5) | 528 (68.7) | 0.962 |  | 0.004 |
| X.4 | Yes | 485 (31.5) | 241 (31.3) |  |  |  |
| drink.... | No | 900 (58.5) | 449 (58.4) | 0.988 |  | 0.003 |
| X.5 | Yes | 638 (41.5) | 320 (41.6) |  |  |  |
| social_score..mean..SD.. |  | 1.31 (0.65) | 1.28 (0.62) | 0.380 |  | 0.039 |
| comorb_n..mean..SD.. |  | 1.25 (1.08) | 1.27 (1.08) | 0.585 |  | 0.024 |
| annual_change..mean..SD.. |  | -0.18 (0.98) | -0.20 (0.89) | 0.611 |  | 0.025 |
| residence.... | Urban | 586 (38.1) | 304 (39.5) | 0.535 |  | 0.029 |
| X.6 | Rural | 952 (61.9) | 465 (60.5) |  |  |  |
| *PSM: 1:2 nearest-neighbor matching without replacement. All SMD < 0.1 after matching. Values are mean (SD) or n (%).* | | | | | | |

Table S4. ELSA — PSM-matched baseline characteristics by incident stroke status.

| **V1** | **level** | **No_Stroke** | **Stroke** | **p** | **test** | **SMD** |
| --- | --- | --- | --- | --- | --- | --- |
| n |  | 612 | 306 |  |  |  |
| age..mean..SD.. |  | 69.04 (9.51) | 69.43 (9.46) | 0.550 |  | 0.042 |
| sex.... | Male | 310 (50.7) | 156 (51.0) | 0.981 |  | 0.007 |
| X | Female | 302 (49.3) | 150 (49.0) |  |  |  |
| bmi..mean..SD.. |  | 27.86 (5.00) | 27.80 (4.76) | 0.858 |  | 0.013 |
| education.... | 1_Below_upper_secondary | 301 (49.2) | 139 (45.4) | 0.485 |  | 0.084 |
| X.1 | 2_Upper_secondary | 245 (40.0) | 128 (41.8) |  |  |  |
| X.2 | 3_Tertiary | 66 (10.8) | 39 (12.7) |  |  |  |
| marital.... | Not_married | 172 (28.1) | 92 (30.1) | 0.588 |  | 0.043 |
| X.3 | Married | 440 (71.9) | 214 (69.9) |  |  |  |
| smoke.... | No | 508 (83.0) | 259 (84.6) | 0.593 |  | 0.044 |
| X.4 | Yes | 104 (17.0) | 47 (15.4) |  |  |  |
| drink.... | No | 91 (14.9) | 37 (12.1) | 0.296 |  | 0.081 |
| X.5 | Yes | 521 (85.1) | 269 (87.9) |  |  |  |
| social_score..mean..SD.. |  | 2.25 (1.15) | 2.18 (1.17) | 0.355 |  | 0.065 |
| comorb_n..mean..SD.. |  | 1.36 (1.07) | 1.35 (1.06) | 0.845 |  | 0.014 |
| annual_change..mean..SD.. |  | -0.21 (0.75) | -0.40 (0.83) | 0.001 |  | 0.239 |
| *PSM: 1:2 nearest-neighbor matching without replacement. All SMD < 0.1 after matching. Values are mean (SD) or n (%).* | | | | | | |

Table S5. CHARLS — Full sample annualized change model coefficients.

| **Study** | **Model** | **Term** | **Beta** | **SE** | **P_value** | **CI_low** | **CI_high** |
| --- | --- | --- | --- | --- | --- | --- | --- |
| CHARLS | A1_AnnualChange_Crude | (Intercept) | -0.160555061076229 | 0.010028533083364 | 5.99327950538639e-57 | -0.180213116972193 | -0.140897005180265 |
| CHARLS | A1_AnnualChange_Crude | incident_stroke | -0.0389404154186935 | 0.0391991228974041 | 0.320540154662604 | -0.11577902606936 | 0.0378981952319733 |
| CHARLS | A2_AnnualChange_Adjusted | (Intercept) | -0.246730179829347 | 0.0747264995808545 | 0.000964278866228236 | -0.393210017783784 | -0.100250341874911 |
| CHARLS | A2_AnnualChange_Adjusted | incident_stroke | -0.0227432878569713 | 0.0390042671944631 | 0.55984150272455 | -0.099199950088214 | 0.0537133743742714 |
| CHARLS | A2_AnnualChange_Adjusted | age_c | -0.0176894516879409 | 0.00120278493915712 | 1.96537623171492e-48 | -0.0200471660726638 | -0.015331737303218 |
| CHARLS | A2_AnnualChange_Adjusted | sexFemale | -0.0566789735313322 | 0.0262261439034745 | 0.0307075548064331 | -0.108087795444555 | -0.00527015161810918 |
| CHARLS | A2_AnnualChange_Adjusted | bmi | 0.00524322462036283 | 0.00264152312280032 | 0.0471814472535076 | 6.52772904196249e-05 | 0.010421171950306 |
| CHARLS | A2_AnnualChange_Adjusted | residenceRural | -0.0931687408070253 | 0.0202510811433149 | 4.26546081065939e-06 | -0.132865168458708 | -0.0534723131553424 |
| CHARLS | A2_AnnualChange_Adjusted | education2_Upper_secondary | 0.0747037651630311 | 0.0325173387363762 | 0.0216202645627618 | 0.0109628628655782 | 0.138444667460484 |
| CHARLS | A2_AnnualChange_Adjusted | education3_Tertiary | 0.0313301452220567 | 0.0798247950474843 | 0.69470764059563 | -0.125143436557992 | 0.187803727002105 |
| CHARLS | A2_AnnualChange_Adjusted | maritalMarried | 0.0301891596250257 | 0.032443298778607 | 0.352125127787905 | -0.033406608602492 | 0.0937849278525434 |
| CHARLS | A2_AnnualChange_Adjusted | smokeYes | -0.0373545450074218 | 0.0252794157844862 | 0.139530095791137 | -0.086907578381985 | 0.0121984883671413 |
| CHARLS | A2_AnnualChange_Adjusted | drinkYes | -0.0237175192596688 | 0.0232385448921341 | 0.307464568057935 | -0.0692700114704619 | 0.0218349729511243 |
| CHARLS | A2_AnnualChange_Adjusted | comorb_n | 0.0291155978578914 | 0.010730602840587 | 0.00667316422962952 | 0.00808133325217565 | 0.0501498624636072 |
| CHARLS | A3_AnnualChange_Interaction | (Intercept) | -0.236968275048774 | 0.0750008659624335 | 0.00158511939503256 | -0.383985933399313 | -0.0899506166982354 |
| CHARLS | A3_AnnualChange_Interaction | incident_stroke | -0.0248696524579902 | 0.047196047431323 | 0.598245053242102 | -0.11738394929597 | 0.0676446443799892 |
| CHARLS | A3_AnnualChange_Interaction | social_highHigh | -0.0353014805131948 | 0.0236235566703964 | 0.135121471601956 | -0.0816086789571772 | 0.0110057179307876 |
| CHARLS | A3_AnnualChange_Interaction | age_c | -0.01722046430662 | 0.00124168884039569 | 2.58279719796762e-43 | -0.019654438679748 | -0.014786489933492 |
| CHARLS | A3_AnnualChange_Interaction | sexFemale | -0.0538453563345578 | 0.0262945500464644 | 0.0406092600058121 | -0.105388270214359 | -0.00230244245475618 |
| CHARLS | A3_AnnualChange_Interaction | bmi | 0.00516352629509113 | 0.0026421637660122 | 0.0506973685561443 | -1.5676969739439e-05 | 0.0103427295599217 |
| CHARLS | A3_AnnualChange_Interaction | residenceRural | -0.0915900973022459 | 0.020277387853071 | 6.351813136922e-06 | -0.13133809276029 | -0.0518421018442022 |
| CHARLS | A3_AnnualChange_Interaction | education2_Upper_secondary | 0.073007434677061 | 0.0325360333050757 | 0.0248620266358645 | 0.00922988534952025 | 0.136784984004602 |
| CHARLS | A3_AnnualChange_Interaction | education3_Tertiary | 0.028469575632493 | 0.0798491618386559 | 0.721442633227534 | -0.128051774409683 | 0.184990925674669 |
| CHARLS | A3_AnnualChange_Interaction | maritalMarried | 0.028518547824521 | 0.0324616044984797 | 0.379677392655324 | -0.0351131052027685 | 0.0921502008518104 |
| CHARLS | A3_AnnualChange_Interaction | smokeYes | -0.0363637312739812 | 0.0252908672335411 | 0.150517764451659 | -0.0859392132449754 | 0.0132117506970131 |
| *Coefficients with \|t\| > 1.96 are significant at p < 0.05. Full table available in results_stroke_cog_final.xlsx.* | | | | | | | |

Table S6. CHARLS — Full sample linear mixed-effects model coefficients.

| **Study** | **Model** | **Term** | **Beta** | **SE** | **df** | **P_value** | **AIC** | **BIC** | **N_obs** | **N_ID** |
| --- | --- | --- | --- | --- | --- | --- | --- | --- | --- | --- |
| CHARLS | B1_LMER_RI | (Intercept) | 9.54086645603715 | 0.224282224388949 | 11072.7680893362 | 0 | 190464.889197254 | 190600.531212398 | 35512 | 11421 |
| CHARLS | B1_LMER_RI | incident_stroke | -0.389165368681583 | 0.116939363399379 | 11016.7760594996 | 0.000877822639690027 | 190464.889197254 | 190600.531212398 | 35512 | 11421 |
| CHARLS | B1_LMER_RI | time_c | -0.112885527227445 | 0.0066550980801262 | 25825.0826112002 | 3.47997271768051e-64 | 190464.889197254 | 190600.531212398 | 35512 | 11421 |
| CHARLS | B1_LMER_RI | age_c | -0.145026112162556 | 0.00361009516183214 | 11432.3757475934 | 0 | 190464.889197254 | 190600.531212398 | 35512 | 11421 |
| CHARLS | B1_LMER_RI | sexFemale | -1.05380819398693 | 0.0788177711071555 | 10962.6744617673 | 1.87221400294809e-40 | 190464.889197254 | 190600.531212398 | 35512 | 11421 |
| CHARLS | B1_LMER_RI | bmi | 0.0723745370072135 | 0.007941951741372 | 11046.4500725837 | 9.39988887020549e-20 | 190464.889197254 | 190600.531212398 | 35512 | 11421 |
| CHARLS | B1_LMER_RI | residenceRural | -1.32402250137856 | 0.0614806131171571 | 10891.5970735439 | 8.97429058788861e-101 | 190464.889197254 | 190600.531212398 | 35512 | 11421 |
| CHARLS | B1_LMER_RI | education2_Upper_secondary | 2.65677774126112 | 0.100783899741875 | 10278.9072733003 | 3.01176995311974e-148 | 190464.889197254 | 190600.531212398 | 35512 | 11421 |
| CHARLS | B1_LMER_RI | education3_Tertiary | 3.61747855994905 | 0.252553040473088 | 10720.7554467331 | 4.14446863853668e-46 | 190464.889197254 | 190600.531212398 | 35512 | 11421 |
| CHARLS | B1_LMER_RI | maritalMarried | 0.529787801606677 | 0.0965847480733956 | 11603.7880656188 | 4.21632051598572e-08 | 190464.889197254 | 190600.531212398 | 35512 | 11421 |
| CHARLS | B1_LMER_RI | smokeYes | -0.229142390013196 | 0.0767738363563421 | 10818.8628164554 | 0.00284545266303405 | 190464.889197254 | 190600.531212398 | 35512 | 11421 |
| CHARLS | B1_LMER_RI | drinkYes | -0.171345364054347 | 0.0699441608778756 | 10946.0171144761 | 0.0143112927914882 | 190464.889197254 | 190600.531212398 | 35512 | 11421 |
| CHARLS | B1_LMER_RI | comorb_n | -0.00656934602611802 | 0.0323404858598172 | 11097.6562235595 | 0.839036588898836 | 190464.889197254 | 190600.531212398 | 35512 | 11421 |
| CHARLS | B1_LMER_RI | incident_stroke:time_c | -0.0658685658611461 | 0.0256505345244749 | 25782.8555752386 | 0.0102366058313129 | 190464.889197254 | 190600.531212398 | 35512 | 11421 |
| CHARLS | B2_LMER_RS | (Intercept) | 9.59133802183893 | 0.220547217677268 | 11044.1805297852 | 0 | 189897.292843374 | 190049.890110412 | 35512 | 11421 |
| CHARLS | B2_LMER_RS | incident_stroke | -0.400864605742987 | 0.117372710208109 | 10917.3004918477 | 0.000639405769029685 | 189897.292843374 | 190049.890110412 | 35512 | 11421 |
| CHARLS | B2_LMER_RS | time_c | -0.133385618075609 | 0.00730349208986742 | 8806.46476954734 | 3.59993137329799e-73 | 189897.292843374 | 190049.890110412 | 35512 | 11421 |
| CHARLS | B2_LMER_RS | age_c | -0.13534884477274 | 0.00353607533032136 | 11233.2676351869 | 2.02510147404903e-301 | 189897.292843374 | 190049.890110412 | 35512 | 11421 |
| CHARLS | B2_LMER_RS | sexFemale | -1.02232488391701 | 0.0775281477805971 | 10946.154771678 | 2.09480794520841e-39 | 189897.292843374 | 190049.890110412 | 35512 | 11421 |
| CHARLS | B2_LMER_RS | bmi | 0.0687612581325737 | 0.00780409252741018 | 10993.2912681925 | 1.42758997838456e-18 | 189897.292843374 | 190049.890110412 | 35512 | 11421 |
| CHARLS | B2_LMER_RS | residenceRural | -1.26521233144628 | 0.0604311635218206 | 10852.9663041685 | 1.89487612512071e-95 | 189897.292843374 | 190049.890110412 | 35512 | 11421 |
| CHARLS | B2_LMER_RS | education2_Upper_secondary | 2.58164647932836 | 0.0994154487857276 | 10381.2172628624 | 4.23412439537546e-144 | 189897.292843374 | 190049.890110412 | 35512 | 11421 |
| CHARLS | B2_LMER_RS | education3_Tertiary | 3.58467576655201 | 0.247871480637179 | 10636.0673630946 | 5.88110839900367e-47 | 189897.292843374 | 190049.890110412 | 35512 | 11421 |
| CHARLS | B2_LMER_RS | maritalMarried | 0.494209252080328 | 0.0946355082511107 | 11395.5447176181 | 1.79887670924878e-07 | 189897.292843374 | 190049.890110412 | 35512 | 11421 |
| CHARLS | B2_LMER_RS | smokeYes | -0.193877125421831 | 0.0755220956966999 | 10805.8859579165 | 0.010266933059575 | 189897.292843374 | 190049.890110412 | 35512 | 11421 |
| *Coefficients with \|t\| > 1.96 are significant at p < 0.05. Full table available in results_stroke_cog_final.xlsx.* | | | | | | | | | | |

Table S7. ELSA — Full sample annualized change model coefficients.

| **Study** | **Model** | **Term** | **Beta** | **SE** | **P_value** | **CI_low** | **CI_high** |
| --- | --- | --- | --- | --- | --- | --- | --- |
| ELSA | A1_AnnualChange_Crude | (Intercept) | -0.172100600343227 | 0.00896011289898685 | 1.01331100102945e-79 | -0.189665828792054 | -0.1545353718944 |
| ELSA | A1_AnnualChange_Crude | incident_stroke | -0.229335188484288 | 0.03867821072067 | 3.22058368992791e-09 | -0.305159189248527 | -0.15351118772005 |
| ELSA | A2_AnnualChange_Adjusted | (Intercept) | -0.155751811695456 | 0.0621866872701216 | 0.0122872767380961 | -0.277661406761929 | -0.0338422166289826 |
| ELSA | A2_AnnualChange_Adjusted | incident_stroke | -0.145609015189258 | 0.0379103791233157 | 0.000123929176163203 | -0.219927799009737 | -0.0712902313687793 |
| ELSA | A2_AnnualChange_Adjusted | age_c | -0.0160788784009818 | 0.00103093756177602 | 9.85268793104843e-54 | -0.0180999087245462 | -0.0140578480774174 |
| ELSA | A2_AnnualChange_Adjusted | sexFemale | -0.00550523830514682 | 0.0177147552548678 | 0.755985836903539 | -0.0402329064705521 | 0.0292224298602584 |
| ELSA | A2_AnnualChange_Adjusted | bmi | 0.000904884308863117 | 0.00175358085530013 | 0.605859884137847 | -0.0025328021377129 | 0.00434257075543913 |
| ELSA | A2_AnnualChange_Adjusted | education2_Upper_secondary | -0.0228029327641496 | 0.0196173971698431 | 0.245128589019977 | -0.0612605038331073 | 0.0156546383048081 |
| ELSA | A2_AnnualChange_Adjusted | education3_Tertiary | -0.0505205077010478 | 0.026713338984203 | 0.0586471856480214 | -0.102888827696802 | 0.00184781229470634 |
| ELSA | A2_AnnualChange_Adjusted | maritalMarried | 0.0348370567317192 | 0.0202276331289348 | 0.0850784346445465 | -0.00481680926680643 | 0.0744909227302447 |
| ELSA | A2_AnnualChange_Adjusted | smokeYes | -0.0303710846719215 | 0.0261455077826061 | 0.24543950451871 | -0.081626239215525 | 0.0208840698716821 |
| ELSA | A2_AnnualChange_Adjusted | drinkYes | -0.0286263640359278 | 0.0289250839113327 | 0.322376229208837 | -0.085330546581901 | 0.0280778185100453 |
| ELSA | A2_AnnualChange_Adjusted | comorb_n | -0.0210496384822324 | 0.00918346587640901 | 0.0219348496484361 | -0.0390527297457494 | -0.00304654721871526 |
| ELSA | A3_AnnualChange_Interaction | (Intercept) | -0.172322919384653 | 0.0630289482788176 | 0.00627583066425427 | -0.295883676100809 | -0.0487621626684971 |
| ELSA | A3_AnnualChange_Interaction | incident_stroke | -0.107262639053334 | 0.071874389584525 | 0.135659607445617 | -0.248163831411645 | 0.0336385533049773 |
| ELSA | A3_AnnualChange_Interaction | social_highHigh | 0.031715933043268 | 0.0191431163373271 | 0.0976190973338101 | -0.00581186973843362 | 0.0692437358249696 |
| ELSA | A3_AnnualChange_Interaction | age_c | -0.0160214564563887 | 0.00103280892545342 | 3.46357124716735e-53 | -0.0180461555170124 | -0.013996757395765 |
| ELSA | A3_AnnualChange_Interaction | sexFemale | -0.00754385969566033 | 0.0177586089527073 | 0.670998435472584 | -0.0423575004170025 | 0.0272697810256818 |
| ELSA | A3_AnnualChange_Interaction | bmi | 0.000753100607854946 | 0.0017558225154626 | 0.668000497779917 | -0.00268898060394175 | 0.00419518181965164 |
| ELSA | A3_AnnualChange_Interaction | education2_Upper_secondary | -0.0222358180697768 | 0.0196382327602512 | 0.257567883195541 | -0.0607342377116626 | 0.016262601572109 |
| ELSA | A3_AnnualChange_Interaction | education3_Tertiary | -0.0466005879220482 | 0.0268555358537865 | 0.082753855458837 | -0.0992476718846869 | 0.00604649604059053 |
| ELSA | A3_AnnualChange_Interaction | maritalMarried | 0.0334174481540087 | 0.020243806778788 | 0.098844991607859 | -0.00626812732692194 | 0.0731030236349394 |
| ELSA | A3_AnnualChange_Interaction | smokeYes | -0.0300603683857847 | 0.0261455972266283 | 0.250303567721586 | -0.0813157021068414 | 0.0211949653352719 |
| ELSA | A3_AnnualChange_Interaction | drinkYes | -0.0282485421121386 | 0.0289346983061875 | 0.328964245552657 | -0.084971576776319 | 0.0284744925520417 |
| ELSA | A3_AnnualChange_Interaction | comorb_n | -0.0213339969460799 | 0.00918435722034715 | 0.0202216445591753 | -0.0393388369297255 | -0.0033291569624343 |
| *Coefficients with \|t\| > 1.96 are significant at p < 0.05. Full table available in results_stroke_cog_final.xlsx.* | | | | | | | |

Table S8. ELSA — Full sample linear mixed-effects model coefficients.

| **Study** | **Model** | **Term** | **Beta** | **SE** | **df** | **P_value** | **AIC** | **BIC** | **N_obs** | **N_ID** |
| --- | --- | --- | --- | --- | --- | --- | --- | --- | --- | --- |
| ELSA | B1_LMER_RI | (Intercept) | 12.5840752519592 | 0.243115203025447 | 5594.52257748163 | 0 | 156623.309287531 | 156748.985069062 | 32157 | 5713 |
| ELSA | B1_LMER_RI | incident_stroke | -0.725411062653353 | 0.148028816330487 | 5564.60812572931 | 9.83170689813675e-07 | 156623.309287531 | 156748.985069062 | 32157 | 5713 |
| ELSA | B1_LMER_RI | time_c | -0.10794782299837 | 0.00361016948644627 | 27627.0874828525 | 2.30502896864699e-193 | 156623.309287531 | 156748.985069062 | 32157 | 5713 |
| ELSA | B1_LMER_RI | age_c | -0.165400800477746 | 0.00406680029503189 | 5788.00904245644 | 2.58626567365934e-318 | 156623.309287531 | 156748.985069062 | 32157 | 5713 |
| ELSA | B1_LMER_RI | sexFemale | 1.1193800673816 | 0.0690942794559099 | 5555.93472339889 | 1.0292635339342e-57 | 156623.309287531 | 156748.985069062 | 32157 | 5713 |
| ELSA | B1_LMER_RI | bmi | -0.0300739576812111 | 0.00683241336173218 | 5530.58327555834 | 1.09447809343404e-05 | 156623.309287531 | 156748.985069062 | 32157 | 5713 |
| ELSA | B1_LMER_RI | education2_Upper_secondary | 1.30843083499341 | 0.0766129028593549 | 5583.52866543878 | 8.74365938202728e-64 | 156623.309287531 | 156748.985069062 | 32157 | 5713 |
| ELSA | B1_LMER_RI | education3_Tertiary | 2.40031269640411 | 0.10392220343185 | 5502.05461417358 | 9.80232438993294e-113 | 156623.309287531 | 156748.985069062 | 32157 | 5713 |
| ELSA | B1_LMER_RI | maritalMarried | 0.231846239927934 | 0.0789950438871088 | 5582.28184772392 | 0.00334965027815785 | 156623.309287531 | 156748.985069062 | 32157 | 5713 |
| ELSA | B1_LMER_RI | smokeYes | -0.479084529064969 | 0.102503222674731 | 5644.53260344579 | 3.02479234276835e-06 | 156623.309287531 | 156748.985069062 | 32157 | 5713 |
| ELSA | B1_LMER_RI | drinkYes | 0.725428071162891 | 0.113754562374968 | 5725.28532775772 | 1.94559588915094e-10 | 156623.309287531 | 156748.985069062 | 32157 | 5713 |
| ELSA | B1_LMER_RI | comorb_n | -0.0423997462371595 | 0.036021429513743 | 5661.15855108544 | 0.239217029163274 | 156623.309287531 | 156748.985069062 | 32157 | 5713 |
| ELSA | B1_LMER_RI | incident_stroke:time_c | -0.177681632932549 | 0.0158939837970161 | 27710.1308139109 | 5.94781167284349e-29 | 156623.309287531 | 156748.985069062 | 32157 | 5713 |
| ELSA | B2_LMER_RS | (Intercept) | 12.5234254537248 | 0.241458063728209 | 5645.93801562762 | 0 | 156027.881021474 | 156170.313573875 | 32157 | 5713 |
| ELSA | B2_LMER_RS | incident_stroke | -0.81495449079018 | 0.150914315646376 | 5303.84763350398 | 6.9499879378067e-08 | 156027.881021474 | 156170.313573875 | 32157 | 5713 |
| ELSA | B2_LMER_RS | time_c | -0.116240179128765 | 0.00446005568281936 | 4046.69501762965 | 1.47417873532583e-138 | 156027.881021474 | 156170.313573875 | 32157 | 5713 |
| ELSA | B2_LMER_RS | age_c | -0.154602696575837 | 0.0040181919123712 | 5731.97655351995 | 2.62394139665807e-288 | 156027.881021474 | 156170.313573875 | 32157 | 5713 |
| ELSA | B2_LMER_RS | sexFemale | 1.11766014336313 | 0.0686105135157206 | 5602.30812318719 | 2.50580012703501e-58 | 156027.881021474 | 156170.313573875 | 32157 | 5713 |
| ELSA | B2_LMER_RS | bmi | -0.0290173454179599 | 0.00678904930029927 | 5589.2010111961 | 1.95068068412897e-05 | 156027.881021474 | 156170.313573875 | 32157 | 5713 |
| ELSA | B2_LMER_RS | education2_Upper_secondary | 1.2978488211201 | 0.076020676345412 | 5615.95697477518 | 9.46726044995844e-64 | 156027.881021474 | 156170.313573875 | 32157 | 5713 |
| ELSA | B2_LMER_RS | education3_Tertiary | 2.3890504702876 | 0.103297603034609 | 5561.61416011102 | 4.53830968516688e-113 | 156027.881021474 | 156170.313573875 | 32157 | 5713 |
| ELSA | B2_LMER_RS | maritalMarried | 0.2292419129249 | 0.0783798433417021 | 5613.80571835053 | 0.00346106310689377 | 156027.881021474 | 156170.313573875 | 32157 | 5713 |
| ELSA | B2_LMER_RS | smokeYes | -0.464500658822675 | 0.101608465277288 | 5659.64432054835 | 4.94606222469136e-06 | 156027.881021474 | 156170.313573875 | 32157 | 5713 |
| ELSA | B2_LMER_RS | drinkYes | 0.745571425401089 | 0.112606646030362 | 5711.42602905018 | 3.89326713360247e-11 | 156027.881021474 | 156170.313573875 | 32157 | 5713 |
| ELSA | B2_LMER_RS | comorb_n | -0.0405635067425389 | 0.0356863685280842 | 5665.0209982018 | 0.255725723745815 | 156027.881021474 | 156170.313573875 | 32157 | 5713 |
| *Coefficients with \|t\| > 1.96 are significant at p < 0.05. Full table available in results_stroke_cog_final.xlsx.* | | | | | | | | | | |

Table S9. CHARLS — PSM-matched annualized change model coefficients.

| **Study** | **Model** | **Term** | **Beta** | **SE** | **P_value** | **CI_low** | **CI_high** |
| --- | --- | --- | --- | --- | --- | --- | --- |
| CHARLS_PSM | A1_AnnualChange_Crude | (Intercept) | -0.175783855944223 | 0.0267947451974813 | 6.91398786269778e-11 | -0.228334351890914 | -0.123233359997531 |
| CHARLS_PSM | A1_AnnualChange_Crude | incident_stroke | -0.0237116205506987 | 0.046545796565418 | 0.610513594414599 | -0.114998351427186 | 0.0675751103257885 |
| CHARLS_PSM | A2_AnnualChange_Adjusted | (Intercept) | -0.13091306581312 | 0.16465785897386 | 0.426678617297986 | -0.453845109727612 | 0.192018978101371 |
| CHARLS_PSM | A2_AnnualChange_Adjusted | incident_stroke | -0.0304516513856994 | 0.0457326736181694 | 0.505581236098601 | -0.12014397373962 | 0.059240670968221 |
| CHARLS_PSM | A2_AnnualChange_Adjusted | age_c | -0.0203957188607818 | 0.00278507076511469 | 3.5771600126892e-13 | -0.0258578850776264 | -0.0149335526439372 |
| CHARLS_PSM | A2_AnnualChange_Adjusted | sexFemale | -0.0895041654359478 | 0.058469710210312 | 0.125992964927274 | -0.204176754664147 | 0.0251684237922508 |
| CHARLS_PSM | A2_AnnualChange_Adjusted | bmi | 0.00362292221223979 | 0.00556763851066426 | 0.515312995780041 | -0.007296501222698 | 0.0145423456471776 |
| CHARLS_PSM | A2_AnnualChange_Adjusted | residenceRural | -0.0564467904375139 | 0.045108154848473 | 0.210957330149778 | -0.144914287423135 | 0.0320207065481072 |
| CHARLS_PSM | A2_AnnualChange_Adjusted | education2_Upper_secondary | 0.163391524240414 | 0.0780651735152485 | 0.0364820404461383 | 0.0102877112859357 | 0.316495337194892 |
| CHARLS_PSM | A2_AnnualChange_Adjusted | education3_Tertiary | 0.0298280741618588 | 0.261956707190522 | 0.90935587723556 | -0.483929455526262 | 0.543585603849979 |
| CHARLS_PSM | A2_AnnualChange_Adjusted | maritalMarried | -0.0362927489030632 | 0.0680474651003165 | 0.593858296680324 | -0.16974952482005 | 0.0971640270139238 |
| CHARLS_PSM | A2_AnnualChange_Adjusted | smokeYes | -0.0931185584726783 | 0.0552406406219989 | 0.0920221275491289 | -0.201458197372271 | 0.0152210804269142 |
| CHARLS_PSM | A2_AnnualChange_Adjusted | drinkYes | 0.0199783649347654 | 0.0520649423943873 | 0.701229441425067 | -0.0821329971860003 | 0.122089727055531 |
| CHARLS_PSM | A2_AnnualChange_Adjusted | comorb_n | 0.0133159205289978 | 0.020902142419956 | 0.524163524015498 | -0.0276780023601597 | 0.0543098434181553 |
| CHARLS_PSM | A3_AnnualChange_Interaction | (Intercept) | -0.0969189053240831 | 0.16684333675528 | 0.561379349289153 | -0.42413740123618 | 0.230299590588014 |
| CHARLS_PSM | A3_AnnualChange_Interaction | incident_stroke | -0.0497739501197638 | 0.0558219270740264 | 0.37269199409804 | -0.159253701432036 | 0.0597058011925083 |
| CHARLS_PSM | A3_AnnualChange_Interaction | social_highHigh | -0.0721176153993546 | 0.0572154673736378 | 0.207661768578253 | -0.184330422581281 | 0.0400951917825719 |
| CHARLS_PSM | A3_AnnualChange_Interaction | age_c | -0.0197368176795148 | 0.00284454030060076 | 5.44079555103093e-12 | -0.0253156212255573 | -0.0141580141334722 |
| CHARLS_PSM | A3_AnnualChange_Interaction | sexFemale | -0.0854650959680667 | 0.0586609273447742 | 0.145302326089161 | -0.200512785542391 | 0.0295825936062571 |
| CHARLS_PSM | A3_AnnualChange_Interaction | bmi | 0.00322940810312235 | 0.00557894557753937 | 0.562755740422099 | -0.00771219865301386 | 0.0141710148592586 |
| CHARLS_PSM | A3_AnnualChange_Interaction | residenceRural | -0.0531480934997031 | 0.0451903721704797 | 0.239707138429655 | -0.141776898814365 | 0.0354807118149586 |
| CHARLS_PSM | A3_AnnualChange_Interaction | education2_Upper_secondary | 0.163611459672485 | 0.0780736658064078 | 0.0362513789628176 | 0.0104908856516708 | 0.3167320336933 |
| CHARLS_PSM | A3_AnnualChange_Interaction | education3_Tertiary | 0.029159728781496 | 0.261985745903054 | 0.911388222357246 | -0.484655107291623 | 0.542974564854615 |
| CHARLS_PSM | A3_AnnualChange_Interaction | maritalMarried | -0.0415921626497516 | 0.0681835780510202 | 0.541934095888601 | -0.175315979792565 | 0.0921316544930619 |
| CHARLS_PSM | A3_AnnualChange_Interaction | smokeYes | -0.0934105439073712 | 0.0552654703093971 | 0.0911532588502487 | -0.201798954389957 | 0.0149778665752147 |
| *Coefficients with \|t\| > 1.96 are significant at p < 0.05. Full table available in results_stroke_cog_final.xlsx.* | | | | | | | |

Table S10. CHARLS — PSM-matched linear mixed-effects model coefficients.

| **Study** | **Model** | **Term** | **Beta** | **SE** | **df** | **P_value** | **AIC** | **BIC** | **N_obs** | **N_ID** |
| --- | --- | --- | --- | --- | --- | --- | --- | --- | --- | --- |
| CHARLS_PSM | B1_LMER_RI | (Intercept) | 9.28854354740217 | 0.488492806464733 | 2190.81245396762 | 9.70557093101732e-75 | 37616.2171664652 | 37725.8712412321 | 6998 | 2267 |
| CHARLS_PSM | B1_LMER_RI | incident_stroke | -0.245213587412265 | 0.136162462186825 | 2192.52948523427 | 0.0718576198138299 | 37616.2171664652 | 37725.8712412321 | 6998 | 2267 |
| CHARLS_PSM | B1_LMER_RI | time_c | -0.153127206475721 | 0.0180476374460907 | 5142.78777785478 | 2.79726863938028e-17 | 37616.2171664652 | 37725.8712412321 | 6998 | 2267 |
| CHARLS_PSM | B1_LMER_RI | age_c | -0.146468068436265 | 0.0082171982582029 | 2286.31636165529 | 1.19658301873662e-66 | 37616.2171664652 | 37725.8712412321 | 6998 | 2267 |
| CHARLS_PSM | B1_LMER_RI | sexFemale | -0.9379592885855 | 0.17316481566678 | 2203.74620170682 | 6.73658545903085e-08 | 37616.2171664652 | 37725.8712412321 | 6998 | 2267 |
| CHARLS_PSM | B1_LMER_RI | bmi | 0.0698996264874815 | 0.016532006176319 | 2192.91405016092 | 2.45301947546249e-05 | 37616.2171664652 | 37725.8712412321 | 6998 | 2267 |
| CHARLS_PSM | B1_LMER_RI | residenceRural | -1.20179721849758 | 0.135237956038774 | 2185.20488150096 | 1.2879145064421e-18 | 37616.2171664652 | 37725.8712412321 | 6998 | 2267 |
| CHARLS_PSM | B1_LMER_RI | education2_Upper_secondary | 2.65309370392791 | 0.237877330492796 | 2053.29787756421 | 4.35165768599719e-28 | 37616.2171664652 | 37725.8712412321 | 6998 | 2267 |
| CHARLS_PSM | B1_LMER_RI | education3_Tertiary | 3.49622698721993 | 0.790425055052472 | 2116.50189213082 | 1.02168750109879e-05 | 37616.2171664652 | 37725.8712412321 | 6998 | 2267 |
| CHARLS_PSM | B1_LMER_RI | maritalMarried | 0.561548997667311 | 0.200703379283044 | 2306.20022870953 | 0.00518624623999496 | 37616.2171664652 | 37725.8712412321 | 6998 | 2267 |
| CHARLS_PSM | B1_LMER_RI | smokeYes | -0.199005463665861 | 0.166278630969058 | 2152.164424545 | 0.231508837413039 | 37616.2171664652 | 37725.8712412321 | 6998 | 2267 |
| CHARLS_PSM | B1_LMER_RI | drinkYes | -0.16720406936558 | 0.154356524301404 | 2195.81943935992 | 0.278823979130068 | 37616.2171664652 | 37725.8712412321 | 6998 | 2267 |
| CHARLS_PSM | B1_LMER_RI | comorb_n | -0.000635930174393495 | 0.0620158514029154 | 2233.76673191182 | 0.991819298177093 | 37616.2171664652 | 37725.8712412321 | 6998 | 2267 |
| CHARLS_PSM | B1_LMER_RI | incident_stroke:time_c | -0.0253409723894642 | 0.0308067085495893 | 5109.09724676114 | 0.410785448065783 | 37616.2171664652 | 37725.8712412321 | 6998 | 2267 |
| CHARLS_PSM | B2_LMER_RS | (Intercept) | 9.43236050608752 | 0.479354253187592 | 2187.5094282797 | 1.69584464750185e-79 | 37498.5948441837 | 37621.9556782964 | 6998 | 2267 |
| CHARLS_PSM | B2_LMER_RS | incident_stroke | -0.245536150473754 | 0.136904162945942 | 2160.42384624349 | 0.0730345324597944 | 37498.5948441837 | 37621.9556782964 | 6998 | 2267 |
| CHARLS_PSM | B2_LMER_RS | time_c | -0.173279487652172 | 0.0197431150623754 | 1771.58793406962 | 3.87975224268493e-18 | 37498.5948441837 | 37621.9556782964 | 6998 | 2267 |
| CHARLS_PSM | B2_LMER_RS | age_c | -0.134198337612766 | 0.00803618638920391 | 2251.92520628173 | 4.1134787532045e-59 | 37498.5948441837 | 37621.9556782964 | 6998 | 2267 |
| CHARLS_PSM | B2_LMER_RS | sexFemale | -0.944794054832163 | 0.169934060481754 | 2200.33178696456 | 3.02885266160122e-08 | 37498.5948441837 | 37621.9556782964 | 6998 | 2267 |
| CHARLS_PSM | B2_LMER_RS | bmi | 0.0634005152512871 | 0.0161939828482648 | 2176.66753443305 | 9.31590545205627e-05 | 37498.5948441837 | 37621.9556782964 | 6998 | 2267 |
| CHARLS_PSM | B2_LMER_RS | residenceRural | -1.15889287602894 | 0.132531384831787 | 2174.06698605623 | 4.40386297991867e-18 | 37498.5948441837 | 37621.9556782964 | 6998 | 2267 |
| CHARLS_PSM | B2_LMER_RS | education2_Upper_secondary | 2.48966593615774 | 0.233852275429152 | 2073.621859563 | 8.30315973460488e-26 | 37498.5948441837 | 37621.9556782964 | 6998 | 2267 |
| CHARLS_PSM | B2_LMER_RS | education3_Tertiary | 3.48475667988353 | 0.776783966023167 | 2128.87382092061 | 7.63898624626993e-06 | 37498.5948441837 | 37621.9556782964 | 6998 | 2267 |
| CHARLS_PSM | B2_LMER_RS | maritalMarried | 0.525520277893817 | 0.196130497287735 | 2264.30238004857 | 0.00742788397409606 | 37498.5948441837 | 37621.9556782964 | 6998 | 2267 |
| CHARLS_PSM | B2_LMER_RS | smokeYes | -0.173810232906353 | 0.16310511512347 | 2147.21254726308 | 0.286709273960661 | 37498.5948441837 | 37621.9556782964 | 6998 | 2267 |
| *Coefficients with \|t\| > 1.96 are significant at p < 0.05. Full table available in results_stroke_cog_final.xlsx.* | | | | | | | | | | |

Table S11. ELSA — PSM-matched annualized change model coefficients.

| **Study** | **Model** | **Term** | **Beta** | **SE** | **P_value** | **CI_low** | **CI_high** |
| --- | --- | --- | --- | --- | --- | --- | --- |
| ELSA_PSM | A1_AnnualChange_Crude | (Intercept) | -0.213180180729642 | 0.0313744362325243 | 1.95617157746561e-11 | -0.274754573120584 | -0.1516057883387 |
| ELSA_PSM | A1_AnnualChange_Crude | incident_stroke | -0.188255608097879 | 0.0542532506405855 | 0.000544936275049836 | -0.294731176891191 | -0.0817800393045666 |
| ELSA_PSM | A2_AnnualChange_Adjusted | (Intercept) | -0.358364368304316 | 0.183947811653223 | 0.0517019838763489 | -0.719378804848927 | 0.00265006824029579 |
| ELSA_PSM | A2_AnnualChange_Adjusted | incident_stroke | -0.177028354307263 | 0.053153424304079 | 0.000901949479517784 | -0.28134682025199 | -0.072709888362537 |
| ELSA_PSM | A2_AnnualChange_Adjusted | age_c | -0.0174238639615334 | 0.00297954529283735 | 6.95403815995588e-09 | -0.0232714946312619 | -0.0115762332918049 |
| ELSA_PSM | A2_AnnualChange_Adjusted | sexFemale | 0.0218881908040072 | 0.0522358552074751 | 0.675296542807906 | -0.0806294617066041 | 0.124405843314619 |
| ELSA_PSM | A2_AnnualChange_Adjusted | bmi | 0.011399474179766 | 0.00528891040088668 | 0.0313975361939173 | 0.00101950286534466 | 0.0217794454941874 |
| ELSA_PSM | A2_AnnualChange_Adjusted | education2_Upper_secondary | 0.0150278934642965 | 0.0550185686755372 | 0.784806286463443 | -0.0929510892231119 | 0.123006876151705 |
| ELSA_PSM | A2_AnnualChange_Adjusted | education3_Tertiary | -0.121564037416989 | 0.0849544511571957 | 0.152795303577355 | -0.288294932668356 | 0.0451668578343773 |
| ELSA_PSM | A2_AnnualChange_Adjusted | maritalMarried | -0.00607610537928041 | 0.0600223809677152 | 0.919389804110248 | -0.123875528217622 | 0.111723317459061 |
| ELSA_PSM | A2_AnnualChange_Adjusted | smokeYes | -0.0814837233486736 | 0.0709951429326662 | 0.251380961082473 | -0.22081819703928 | 0.0578507503419326 |
| ELSA_PSM | A2_AnnualChange_Adjusted | drinkYes | -0.0758759229491701 | 0.0742572199039994 | 0.307149593960281 | -0.221612521618524 | 0.0698606757201834 |
| ELSA_PSM | A2_AnnualChange_Adjusted | comorb_n | -0.0164830879985939 | 0.0246814251012223 | 0.504410194184689 | -0.0649226464578895 | 0.0319564704607017 |
| ELSA_PSM | A3_AnnualChange_Interaction | (Intercept) | -0.381575960777334 | 0.191779661315518 | 0.046930193981167 | -0.757962238797122 | -0.00518968275754589 |
| ELSA_PSM | A3_AnnualChange_Interaction | incident_stroke | -0.125108341085716 | 0.102114424698564 | 0.2208280099491 | -0.32551785260942 | 0.0753011704379893 |
| ELSA_PSM | A3_AnnualChange_Interaction | social_highHigh | 0.0343296646250598 | 0.0694493285999194 | 0.621205926019179 | -0.101971411950998 | 0.170630741201118 |
| ELSA_PSM | A3_AnnualChange_Interaction | age_c | -0.017463825452476 | 0.00300060297163278 | 8.1679882547771e-09 | -0.0233528012518956 | -0.0115748496530564 |
| ELSA_PSM | A3_AnnualChange_Interaction | sexFemale | 0.0221897667056868 | 0.052299921204418 | 0.671463405099789 | -0.0804539263107128 | 0.124833459722086 |
| ELSA_PSM | A3_AnnualChange_Interaction | bmi | 0.0114282096572346 | 0.00529396523046016 | 0.0311347422599759 | 0.00103828689174981 | 0.0218181324227195 |
| ELSA_PSM | A3_AnnualChange_Interaction | education2_Upper_secondary | 0.0142132610867136 | 0.0553140402532925 | 0.79727152750576 | -0.0943459343480991 | 0.122772456521526 |
| ELSA_PSM | A3_AnnualChange_Interaction | education3_Tertiary | -0.121440473482847 | 0.0856178403893027 | 0.156418978727673 | -0.289473830384049 | 0.0465928834183539 |
| ELSA_PSM | A3_AnnualChange_Interaction | maritalMarried | -0.00682219297050948 | 0.0600888648205941 | 0.909631631846544 | -0.124752447018922 | 0.111108061077903 |
| ELSA_PSM | A3_AnnualChange_Interaction | smokeYes | -0.0820319341031116 | 0.0710928560597501 | 0.248858991663964 | -0.221558593513062 | 0.0574947253068387 |
| ELSA_PSM | A3_AnnualChange_Interaction | drinkYes | -0.0771312520015288 | 0.0744451475223375 | 0.300441725452574 | -0.223237110153336 | 0.0689746061502783 |
| ELSA_PSM | A3_AnnualChange_Interaction | comorb_n | -0.0169160313440669 | 0.0247137475225273 | 0.493848823848757 | -0.0654191696962679 | 0.0315871070081342 |
| *Coefficients with \|t\| > 1.96 are significant at p < 0.05. Full table available in results_stroke_cog_final.xlsx.* | | | | | | | |

Table S12. ELSA — PSM-matched linear mixed-effects model coefficients.

| **Study** | **Model** | **Term** | **Beta** | **SE** | **df** | **P_value** | **AIC** | **BIC** | **N_obs** | **N_ID** |
| --- | --- | --- | --- | --- | --- | --- | --- | --- | --- | --- |
| ELSA_PSM | B1_LMER_RI | (Intercept) | 13.1706232875706 | 0.625898305871124 | 907.864991973652 | 2.23384617537655e-80 | 24525.4679666256 | 24622.8891125643 | 4889 | 918 |
| ELSA_PSM | B1_LMER_RI | incident_stroke | -0.555621980996765 | 0.179420387904575 | 883.123638057154 | 0.00201827892706283 | 24525.4679666256 | 24622.8891125643 | 4889 | 918 |
| ELSA_PSM | B1_LMER_RI | time_c | -0.146542587183257 | 0.012516568642505 | 4247.72172069112 | 3.48352017300344e-31 | 24525.4679666256 | 24622.8891125643 | 4889 | 918 |
| ELSA_PSM | B1_LMER_RI | age_c | -0.182267270353651 | 0.0101815119452607 | 928.469272681356 | 8.51277884555281e-62 | 24525.4679666256 | 24622.8891125643 | 4889 | 918 |
| ELSA_PSM | B1_LMER_RI | sexFemale | 1.24397149045822 | 0.176950765739298 | 894.472017552221 | 4.09655904739959e-12 | 24525.4679666256 | 24622.8891125643 | 4889 | 918 |
| ELSA_PSM | B1_LMER_RI | bmi | -0.0482624215304495 | 0.0179976694227992 | 910.063508365518 | 0.00745992714883607 | 24525.4679666256 | 24622.8891125643 | 4889 | 918 |
| ELSA_PSM | B1_LMER_RI | education2_Upper_secondary | 1.42272227661271 | 0.186171293695403 | 892.494161660533 | 5.51365680543271e-14 | 24525.4679666256 | 24622.8891125643 | 4889 | 918 |
| ELSA_PSM | B1_LMER_RI | education3_Tertiary | 2.20489170179227 | 0.286083361712687 | 874.677850819661 | 3.48862970822482e-14 | 24525.4679666256 | 24622.8891125643 | 4889 | 918 |
| ELSA_PSM | B1_LMER_RI | maritalMarried | 0.192074945222474 | 0.203307611452201 | 898.127135542773 | 0.345040407794155 | 24525.4679666256 | 24622.8891125643 | 4889 | 918 |
| ELSA_PSM | B1_LMER_RI | smokeYes | -0.565318995235677 | 0.238852207918543 | 872.514820778005 | 0.0181593589601123 | 24525.4679666256 | 24622.8891125643 | 4889 | 918 |
| ELSA_PSM | B1_LMER_RI | drinkYes | 0.548753595106407 | 0.252768176899236 | 910.150802173778 | 0.030190817535213 | 24525.4679666256 | 24622.8891125643 | 4889 | 918 |
| ELSA_PSM | B1_LMER_RI | comorb_n | -0.0367546296026366 | 0.0838476074054632 | 904.333942121857 | 0.661237009467538 | 24525.4679666256 | 24622.8891125643 | 4889 | 918 |
| ELSA_PSM | B1_LMER_RI | incident_stroke:time_c | -0.13885154613291 | 0.0208398171022177 | 4219.07130408279 | 3.03258354597366e-11 | 24525.4679666256 | 24622.8891125643 | 4889 | 918 |
| ELSA_PSM | B2_LMER_RS | (Intercept) | 13.3566359689719 | 0.620878705218029 | 917.244565153403 | 1.97519407205767e-83 | 24440.6099260595 | 24551.0205581234 | 4889 | 918 |
| ELSA_PSM | B2_LMER_RS | incident_stroke | -0.587450463517845 | 0.183768696442699 | 830.293771961842 | 0.00144277087308956 | 24440.6099260595 | 24551.0205581234 | 4889 | 918 |
| ELSA_PSM | B2_LMER_RS | time_c | -0.157579824780477 | 0.0154353866821801 | 612.504361607806 | 1.0534355577231e-22 | 24440.6099260595 | 24551.0205581234 | 4889 | 918 |
| ELSA_PSM | B2_LMER_RS | age_c | -0.170869154193644 | 0.0100491689154249 | 919.072575327781 | 1.392427575956e-56 | 24440.6099260595 | 24551.0205581234 | 4889 | 918 |
| ELSA_PSM | B2_LMER_RS | sexFemale | 1.23238998976506 | 0.175586889102758 | 903.510033737685 | 4.3956676076664e-12 | 24440.6099260595 | 24551.0205581234 | 4889 | 918 |
| ELSA_PSM | B2_LMER_RS | bmi | -0.0563501822766216 | 0.0178368462178404 | 917.225912848042 | 0.00163382591872852 | 24440.6099260595 | 24551.0205581234 | 4889 | 918 |
| ELSA_PSM | B2_LMER_RS | education2_Upper_secondary | 1.36753572804262 | 0.18471322509349 | 900.687036367751 | 3.04310309871976e-13 | 24440.6099260595 | 24551.0205581234 | 4889 | 918 |
| ELSA_PSM | B2_LMER_RS | education3_Tertiary | 2.19915451669409 | 0.284444915378718 | 887.795421412207 | 2.87860909471613e-14 | 24440.6099260595 | 24551.0205581234 | 4889 | 918 |
| ELSA_PSM | B2_LMER_RS | maritalMarried | 0.158412860395821 | 0.201556147116838 | 904.331994876304 | 0.432103222387179 | 24440.6099260595 | 24551.0205581234 | 4889 | 918 |
| ELSA_PSM | B2_LMER_RS | smokeYes | -0.501585253978417 | 0.237661508004811 | 887.175314137331 | 0.0350944256314006 | 24440.6099260595 | 24551.0205581234 | 4889 | 918 |
| ELSA_PSM | B2_LMER_RS | drinkYes | 0.577356032335597 | 0.250312597029186 | 913.315278043739 | 0.0213035207215791 | 24440.6099260595 | 24551.0205581234 | 4889 | 918 |
| ELSA_PSM | B2_LMER_RS | comorb_n | -0.0511174812027048 | 0.0830465017247154 | 907.409260674845 | 0.538360158797823 | 24440.6099260595 | 24551.0205581234 | 4889 | 918 |
| *Coefficients with \|t\| > 1.96 are significant at p < 0.05. Full table available in results_stroke_cog_final.xlsx.* | | | | | | | | | | |

Figure S1. CHARLS — Individual cognitive trajectories by incident stroke status.

Data sources: CHARLS 2011–2018 (Waves 1–4), ELSA 2006–2019 (Waves 3–9).

Figure S2. ELSA — Individual cognitive trajectories by incident stroke status.

Data sources: CHARLS 2011–2018 (Waves 1–4), ELSA 2006–2019 (Waves 3–9).

Figure S3. CHARLS — Cognitive trajectories by stroke and social support level.

Data sources: CHARLS 2011–2018 (Waves 1–4), ELSA 2006–2019 (Waves 3–9).

Figure S4. ELSA — Cognitive trajectories by stroke and family connection level.

Data sources: CHARLS 2011–2018 (Waves 1–4), ELSA 2006–2019 (Waves 3–9).

Figure S5. CHARLS — Waterfall plot of individual annual cognitive change.

Data sources: CHARLS 2011–2018 (Waves 1–4), ELSA 2006–2019 (Waves 3–9).

Figure S6. ELSA — Waterfall plot of individual annual cognitive change.

Data sources: CHARLS 2011–2018 (Waves 1–4), ELSA 2006–2019 (Waves 3–9).

Figure S7. CHARLS — Propensity score density by stroke status.

Data sources: CHARLS 2011–2018 (Waves 1–4), ELSA 2006–2019 (Waves 3–9).

Figure S8. ELSA — Propensity score density by stroke status.

Data sources: CHARLS 2011–2018 (Waves 1–4), ELSA 2006–2019 (Waves 3–9).

Figure S9. CHARLS — PSM-matched cognitive trajectories by stroke status.

Data sources: CHARLS 2011–2018 (Waves 1–4), ELSA 2006–2019 (Waves 3–9).

Figure S10. ELSA — PSM-matched cognitive trajectories by stroke status.

Data sources: CHARLS 2011–2018 (Waves 1–4), ELSA 2006–2019 (Waves 3–9).
